# Analyses between Reproductive Behavior, Genetic Diversity and *Pythium* Responsiveness in *Zingiber* spp. Reveal an Adaptive Significance for Hemiclonality

**DOI:** 10.3389/fpls.2016.01913

**Published:** 2016-12-20

**Authors:** Geethu E. Thomas, Kiran A. Geetha, Lesly Augustine, Sabu Mamiyil, George Thomas

**Affiliations:** ^1^Department of Botany, St. Thomas’ College, ThrissurIndia; ^2^Plant Disease Biology and Biotechnology, Rajiv Gandhi Centre for BiotechnologyThiruvananthapuram, India; ^3^Department of Botany, University of CalicutMalappuram, India

**Keywords:** breeding strategy, disease resistance, genetic diversity, Muller’s ratchet, Red Queen, *Zingiber* spp.

## Abstract

Mode of reproduction is generally considered to have long-range evolutionary implications on population survival. Because sexual reproduction produces genetically diverse genotypes, this mode of reproduction is predicted to positively influence the success potential of offspring in evolutionary arms race with parasites (Red queen) whereas, without segregation and recombination, the obligate asexual multiplication may push a species into extinction due to the steady accumulation of deleterious mutations (Muller’s ratchet). However, the extent of linearity between reproductive strategies, genetic diversity and population fitness, and the contributions of different breeding strategies to population fitness are yet to be understood clearly. Genus *Zingiber* belonging to the pan-tropic family Zingiberaceae represents a good system to study contributions of different breeding behavior on genetic diversity and population fitness, as this genus comprises species with contrasting breeding systems. In this study, we analyzed breeding behavior, amplified fragment length polymorphism diversity and response to the soft-rot pathogen *Pythium aphanidermatum* in 18 natural populations of three wild *Zingiber* spp.: *Z. neesanum, Z. nimmonii*, and *Z. zerumbet*, together with the obligately asexual cultivated congener, ginger (*Z. officinale*). Ginger showed an exceptionally narrow genetic base, and adding to this, all the tested cultivars were uniformly susceptible to soft-rot. Concordant with the postulates of Muller’s ratchet, the background selection may be continuously pushing ginger into the ancestral state, rendering it inefficient in host-pathogen coevolution. *Z. neesanum* and *Z. nimmonii* populations were sexual and genetically diverse; however, contrary to Red Queen expectations, the populations were highly susceptible to soft-rot. *Z. zerumbet* showed a hemiclonal breeding behavior. The populations inhabiting forest understory were large and continuous, sexual and genetically diverse, but were susceptible, whereas populations inhabiting the revenue land were fragmented and monoclonal, but were resistant. It may be possible that, when genetic recombination becomes at a premium due to the genetic constraints imparted by habitat fragmentation or pathogen pressure, Z. zerumbet

## Introduction

In principle, assumptions of Red queen hypothesis and the Muller’s ratchet, the two prominent theories on sex, essentially represent two sides of the same coin. While Red queen postulates that the genetic recombination associated with sexual reproduction is essential for both host and pathogen to develop new variants in order to lock in an evolutionary arms race, the Muller’s ratchet hypothesize the extinction of asexuals by the accumulation of deleterious mutation due to their inability to purge out harmful mutations by sexual selection (Gabriel et al., 1993; Clay and Kover, 1996; Lively and Dybdahl, 2000; Kaiser and Charlesworth, 2010; Morran et al., 2011).

Thus, both the hypotheses imply a linear relationship between sex, genetic diversity, and population survival and many authors argue that genetic diversity buffer natural populations against various biotic stresses (Rice, 2002; Keesing et al., 2010; King and Lively, 2012; Civitello et al., 2015). Supporting the hypotheses partly, empirical studies generally report high genetic diversity in sexuals such as *Oenothera* spp. (Godfrey and Johnson, 2014) and a narrow genetic base in asexuals such as *Ziziphus celata* (Gitzendanner et al., 2012) and *Gagea spathacea* (Pfeiffer et al., 2012). Although, genetic diversity is predicted to safe guard populations from infections (Rice, 2002; Keesing et al., 2010; Civitello et al., 2015), the amount of genetic diversity needed for a population to prevent disease spread is not clear (King and Lively, 2012). Similarly, little is known about how the population genetic structure of a host species influences disease dynamics in natural conditions, because in evolutionary arms race pathogen competes not with species as a whole, but with populations, which are spatially structured and experience selection mosaics (Forde et al., 2004; Laine, 2006). Also, relatively little is understood about the contributions of different breeding strategy on population fitness, although breeding behavior is indicated to have a role in the emergence and fixation of resistance traits in natural populations (Rice, 2002; Koslow and DeAngelis, 2006; Campbell and Kessler, 2013). Occurrence of clonal lineages that are more adaptive than sexual lineages are reported in certain species (Peck et al., 1998; Johnson et al., 2010) rendering it difficult to explain the survival and the adaptive fitness of genetically narrow asexuals solely on the basis of existing theories of sex.

Furthermore, the evolutionary ecologists are anxious to know about how the eco-evolutionary feedbacks will shape the genetic architecture of natural populations to cope with the ongoing ecological degradation caused by habitat fragmentation and climate change (Alsos et al., 2012; Jacquemyn et al., 2012). It is predicted that the ecological degradation will adversely affect breeding behavior of natural populations and trigger disease epidemics; both of which affect global biodiversity critically (Eckert et al., 2010; Giraud et al., 2010; Fisher et al., 2012). The fungal and oomycete pathogens are predicted to account a greater share of the disease catastrophes caused by ecological degradations (Giraud et al., 2010; Fisher et al., 2012). Upsurges of epidemics caused by soil microbiota (Klironomos, 2002), particularly the species of the oomycete necrotroph *Pythium* may critically affect the demography and diversity of an ecosystem (Gómez-Aparicio et al., 2012). Thus, the ongoing habitat fragmentation and climate change may trigger a drastic shift in the reproductive strategy, host resistance and demography in natural populations in the near future.

Therefore, baseline information about how the shift in the trade-offs between breeding strategy, genetic diversity and pathogen resistance affect the fitness and survival of natural populations, especially in species rich tropical ecosystem (Fayle et al., 2015), is extremely important in furthering our understandings about the role of eco-evolutionary feedbacks in the evolution of host defense and to refine the models for predicting population dynamics in the changing climatic scenario (Boots et al., 2009; Garrett et al., 2011). In addition to the interests in understanding eco-evolutionary feedbacks in governing population dynamics, the analysis of host-pathogen interactions in natural habitats is also important in drawing conclusions for designing crop protection strategies against evolving pathogen populations (Zhan et al., 2014) and to successfully incorporate evolutionary principles in crop improvement protocols (Zhan et al., 2015).

The genus *Zingiber*, which belong to the pan-tropic family Zingiberaceae, represents a good system to study the relationship between breeding strategy, genetic diversity, and pathogen resistance. Though not empirically documented, different breeding systems, such as sexuality, clonality, and a combination of both are known to occur among the species of this genus (Kavitha et al., 2010). Similarly, the response to the necrotrophic oomycete *Pythium*, which causes soft rot disease in Zingiberaceae, vary between *Zingiber* species (Kavitha and Thomas, 2007). With the premise of the argument of Red queen and Muller’s ratchet, one would expect relatively higher genetic diversity and pathogen resistance in sexual populations (Clay and Kover, 1996; Lively and Dybdahl, 2000; Morran et al., 2011) and a narrow genetic diversity and predominant pathogen susceptibility in asexual populations (Gabriel et al., 1993; Kaiser and Charlesworth, 2010; Pfeiffer et al., 2012), respectively. However, the relationship between genetic diversity and pathogen responsiveness in those species that comprise both sexually and asexually reproducing populations is relatively less studied and the genetic consequences and adaptive significance of this kind of breeding behavior is not fully explained (Goodwillie et al., 2005; McKey et al., 2010). In this study, we investigated the dynamics of soft-rot disease *vis-à-vis* reproductive strategy and genetic diversity in natural populations of three wild species of genus *Zingiber*: *Z. neesanum* (J. Graham) Ramamoorthy, *Z. nimmonii* (J. Graham) Dalzell, and *Z. zerumbet* (L.) Smith in conjunction with the cultivated congener and world renowned spice crop ginger (*Z*. *officinale* Roscoe), which is notoriously asexual (Ramachandran, 1969) and highly susceptible to the soft-rot disease (Le et al., 2014). We analyzed the data sets and tried to draw inferences regarding: (1) level of genetic diversity and nature of molecular differentiation between populations *vis-à-vis* reproductive strategy; (2) pathogen responsiveness *vis-à-vis* reproductive strategy and genetic diversity in *Zingiber* spp.

## Materials and Methods

### Plant Materials and Seed Germination Test

Ginger is an important spice crop in India and in many other South and Southeast Asian countries (Le et al., 2014). In Kerala, ginger is cultivated throughout the geographic area, with the commercial cultivation focusing mostly to eastern highlands, which include the central region of Western Ghats. *Z. nimmonii* and *Z. neesanum* are endemic to South India while *Z. zerumbet* is distributed abundantly in South and Southeast Asian countries (Sabu, 2003). Ginger and the three wild species chosen for the study are rhizomatous perennials and the populations consist of isolated ramets with no underground connections through rhizomes.

**FIGURE 1:**
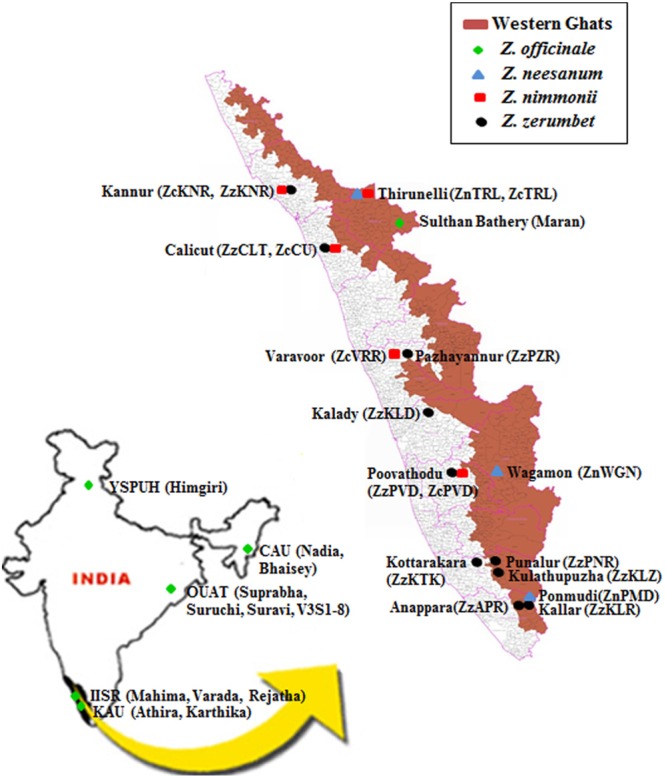
**Map indicating the collecting site of populations of wild *Zingiber* spp. or the cultivars of ginger included in this study.** Parentheses represent the name of the populations or cultivars. YSPUH – Dr. Y. S. Parmer University of Horticulture and Forestry, Solan, Himachal Pradesh; CAU – Central Agricultural University, Imphal, Manipur; OUAT – Orissa University of Agricultural and Technology, Bhubaneswar, Odisha; IISR – Indian Institute of Spice Research, Calicut, Kerala; KAU – Kerala Agriculture University, Thrissur, Kerala.

Geographic origin of the plant materials used in the study is given in **Figure 1**. Altogether, 13 ginger cultivars, consisting of the following 12 released varieties: Mahima, Varada, and Rejatha (Indian Institute of Spices Research, Calicut, Kerala), Nadia and Bhaisey (Central Agricultural University, Imphal, Manipur), Himgiri (Dr. Y. S. Parmer University of Horticulture and Forestry, Solan, Himachal Pradesh), Suprabha, Suravi, and Suruchi, V_3_S_1_-8 (Orissa University of Agriculture and Technology, Bhubaneswar, Odisha), Athira and Karthika (Kerala Agricultural University, Thrissur, Kerala), and Maran, a local cultivar in Kerala, were used in the study (**Figure 1**; Supplementary Table S1). The varieties are developed by clonal selection by the respective research institutions from local cultivars.

Altogether, 18 natural populations comprised of three, five, and 10 populations of *Z. neesanum*, Z. *nimmonii*, and *Z. zerumbet*, respectively were included in the study (**Figure 1**; Supplementary Tables S2a–c). The populations were sampled by the investigating group from 14 sites in Kerala in 2008–2010. Geographic distance between the collecting sites is given in Supplementary Tables S3a–c. In the natural habitats, the rhizome of the wild *Zingiber* spp. sprout after the first monsoon (south-west) shower in late May or early June, tillers (pseudostem) emerge and flowering occurs in September–October. The tillers dry by January with the onset of summer. The *Z. neesanum* populations were collected from forest in the cooler mountain ranges of eastern highlands of Kerala. The *Z. nimmonii* populations except ZnTRL and the *Z. zerumbet* populations, except the three populations from southern Kerala: ZzKLZ, ZzAPR, and ZzKLR, were from unmanaged revenue land (agricultural land). The three south Kerala populations of *Z. zerumbet* occupied the understory of the evergreen forest at the slope or foot hills of the Western Ghats mountain ranges. No *Z. zerumbet* populations were found at the hill-tops (∼1000 m above mean sea level) of Western Ghats. The three south Kerala populations of *Z. zerumbet* occurred in continuous stretches in forest ranges, whereas the other seven populations of *Z. zerumbet* and all the populations of the other two species were occurred in isolated patches.

Ginger never set seeds, although flowers profusely (Ramachandran, 1969). Populations of the wild *Zingiber* spp. were carefully examined for seed set under natural habitats for three consecutive years since 2008, between September and December. Approximately 30–40 randomly selected plants that were minimum 3 m apart were chosen from seed-setting populations in one of the seasons (2009) for seed sampling and the seeds collected from the selected plants were pooled and brought to the laboratory. Damaged, infected or undersized seeds were removed from seed lots and 500 good seeds were used for germination test from each seed-setting population. As many seed as possible were collected if the number of plants in a population was not adequate to yield 500 seeds. Seed germination tests were performed in Petri dishes (9 cm) lined with two sterile filter paper disks, with 20 seeds per Petri dishes. The seeds were stored in room temperature (22°C) for 45 days and the seeds were moistened with deionized sterile water every alternate day. Germinated seeds were tabulated and were transplanted into 10 in. earthen pots in a sand-soil-compost mixture. One-way ANOVA was performed to test the significance of number of seeds germinated between *Zingiber* spp. For seed setting populations, the plants emerged from seeds were used for further studies whereas for non-seed-setting populations, the plants raised from rhizomes were used. The plants were maintained in a wire-mesh net house at Rajiv Gandhi Centre for Biotechnology.

### Pathogen Inoculation

Collar region of the tillers of four months old healthy potted plants with uniform growth were inoculated with a field isolate of *Pythium aphanidermatum* (Edson) Fitzp. (RGCB P117) obtained from the Indian Institute of Spices Research, Kozhikode, Kerala, as described before (Kavitha and Thomas, 2008). The inoculated plants were observed regularly for a period of 30 days and disease symptoms of individuals plants were scored as described elsewhere (Kavitha and Thomas, 2008) using an increasing susceptibility (0–9) scale: 0: no symptoms; 1: up to 25% tiller death; 3: 26–50% tiller death; 5: 51–75% tiller death; 7: >75% tiller death after 25 days post inoculation (dpi); 9: >75% tiller death within 25 dpi. Altogether 812 individuals, comprising of three to six individuals per ginger cultivars (Supplementary Table S1) and 30–60 individuals per natural populations (Supplementary Tables S2a–c), were subjected to pathogen screening. Plants mock inoculated with sterile water were used as control. A representative set of 3–4 plants each from ginger cultivars and natural populations were maintained in pots for two years in order to examine the general performance of plants in the experimental conditions.

### Diversity Analysis of *Zingiber* spp. Using AFLP

Amplified fragment length polymorphism (AFLP) genotyping was performed on altogether 249 individuals, comprising of three individuals each from the 13 ginger cultivars, 10 individuals each from three populations of *Z. neesanum*, five populations of *Z. nimmonii*, and seven of the 10 populations of *Z. zerumbet*, and 20 individuals each from the three south Kerala populations of *Z. zerumbet* (ZzKLZ, ZzAPR, and ZzKLR) (Supplementary Tables S1 and S2a–c). Tender leaf tissues were sampled from each plant one month before pathogen inoculation. Genomic DNA was isolated from 100 mg of the leaf tissues using a GenElute Plant Genomic DNA Purification Kit (Sigma) following the manufacturer’s instructions.

Amplified fragment length polymorphism analysis was carried out by using AFLP Analysis System I (Life Technologies) following the manufacturer’s instructions. Initially, we evaluated 26 primer combinations in a set of altogether 44 individuals selected randomly from the four *Zingiber* spp. (Supplementary Tables S1 and S2a–c) used in the study. Based on the results of initial screening, we chose nine primer combinations that produced fingerprint profiles consisting of relatively higher proportion of discrete and conspicuous bands, which are generally considered as the product of selective amplifications (Bonin et al., 2004). AFLP primer combinations used for genotyping each *Zingiber* species are listed in Supplementary Table S4. In order to further ensure the reliability of genotypes scored based on AFLP profiles, we estimated error rate also by computing the percentage of irreproducible fragments between replicates, according to the method of Bonin et al. (2004). The error rate was estimated by comparing the profiles generated in the 44 individuals between initial evaluation and final fingerprinting using respective primer combinations.

Amplified fragment length polymorphism profiles were independently scrutinized by two persons and the bands that were consistently occurred in both the readings were chosen for further analyses. Bands were scored as either present (1) or absent (0), and the resulting 1/0 matrix was used to compute the genetic diversity parameters and the population genetic characteristics of *Zingiber* spp.

The genetic diversity parameters: percentage of polymorphic bands (PPB), Nei’s genetic diversity (*h*) and Shannon information index (*I*), and the gene flow between populations (*Nm*) were computed using the software POPGENE (Yeh et al., 1999). Significance of difference in *h* between populations was tested by performing one-way ANOVA. Allele richness (Ar) and private alleles (Pa) were evaluated by using the soft ware ADZE (Allelic Diversity AnalyZEr) version 1.0 (Szpiech et al., 2008), which also uses a rarefaction approach to size-correct the uneven sample size between populations within a species by chopping samples down to a standardized size.

Principle coordinate analysis (PCoA) was performed based on pair-wise genetic distance matrix, in order to ordinate relationships among individuals and populations within a species, as implemented in the software program GenAlEx ver. 6.5 (Peakall and Smouse, 2012). In order to understand the pattern of population sub division within *Zingiber* spp. we subjected AFLP data into Bayesian algorithm, as implemented in the software STRUCTURE ver. 2.3.4 (Pritchard et al., 2000). We performed 10 independent iterations for each *K* (number of population genetic clusters) between *K*= 1–10 and identified the optimum number of clusters in the dataset by using the second order statistics (Δ*K*) developed by Evanno et al. (2005) and the *ad hoc* procedure described by Pritchard et al. (2000). Each run was performed using a burn-in period of 10000 with 100000 Markov Chain Monte Carlo (MCMC) replications after burn-in, allowing for admixture and correlated allele frequencies. A UPGMA (unweighted pair group method with arithmetic averages) dendrogram depicting the genetic relationship between 130 *Z. zerumbet* individuals was constructed using the SAHN (sequential, agglomerative, hierarchical, and nested cluster) module of the software NTSYSpc2.02 I based on pair-wise DICE genetic distance. Reliability of the topology of the resulting dendrogram was tested by determining the cophenetic correlation using the COPH and MXCOMP procedures of NTSYSpc.

Molecular differentiation between the populations within a species was tested following analysis of molecular variance (AMOVA) (significance tested with 1023 permutations) using the software ARLEQUIN ver. 3.0 (Excoffier et al., 2005).

## Results

### Seed Set and Seed Germination

All of the *Z. neesanum* and *Z. nimmonii* populations and the three populations of *Z. zerumbet* from forest ranges (ZzKLR, ZzAPR, and ZzKLZ) were flowered profusely in natural habitats and produced seeds. The percentage germination of seeds collected from these populations is given **Table 1**. The percentage ranged from 7.5% (ZnTRL) to 27.4% (ZnWGN) in *Z. neesanum* populations, 13.0% (ZcPVD) to 28.8% (ZcCU) in *Z. nimmonii* populations and 19.6% (ZzAPR) to 46.8% (ZzKLZ) in the three *Z. zerumbet* populations. ANOVA showed no statistical significance (*p*= 0.16889) in seed germination between the *Zingiber* spp. All of the seven *Z. zerumbet* populations sampled from revenue lands (ZzKTK, ZzPNR, ZzPVD, ZzKLD, ZzPZR, ZzCLT, and ZzKNR) were also flowered excessively under natural habitats, but produced no seeds. A few plants in two of the *Z. zerumbet* populations (ZzKTK and ZzPNR) produced a few seeds under experimental conditions, but the seeds were not viable.

**Table 1:** Details of seed germination studies conducted in different populations of the wild species of genus *Zingiber.*

Species/population	No. of seeds planted	No. of seeds germinated	Germination (%)
***Z. neesanum***			
ZnTRL	200	15	7.5
ZnWGN	500	137	27.4
ZnPMD	500	98	19.6
***Z. nimmonii***			
ZcKNR	500	124	24.8
ZcTRL	500	103	20.6
ZcCU	500	144	28.8
ZcVRR	500	143	28.6
ZzPVD	500	65	13.0
***Z. zerumbet***			
ZzKLZ	250	117	46.8
ZzAPR	500	98	19.6
ZzKLR	500	184	36.8

### Response of *Zingiber* spp. to *P. aphanidermatum*

All of the ginger cultivars tested were invariably susceptible to *P. aphanidermatum* and yielded a disease score 9 (Supplementary Table S1). In *Z. neesanum*, plants in ZnTRL and ZnPMD populations were wilted completely within 25 dpi and yielded a score of 9 whereas, of the 42 ZnWGN plants, 37 yielded score 9, while the remaining five plants yielded a score between 3 and 7 (Supplementary Table S2a). In the 300 plants evaluated in total from the five populations of *Z. nimmonii*, score 9 was recorded in 263 plants (87%), ranging from 46 plants in ZcPVD population to all the plants in ZcVRR population. In the remaining 37 plants, score 7 was recorded in 20 plants, followed by score 5 in nine plants and score 3 in six plants. Two of the plants did not show any symptoms (Supplementary Table S2b).

**FIGURE 2:**
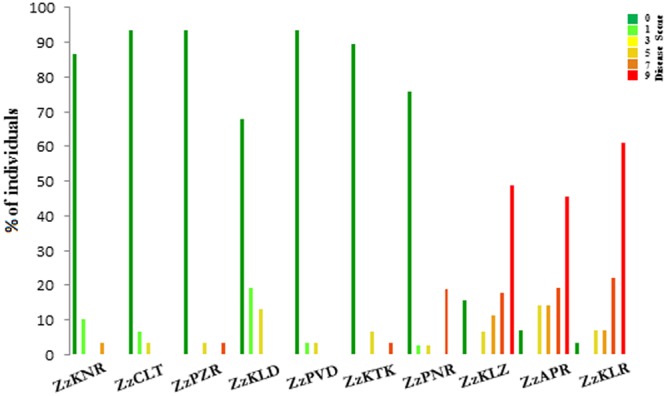
**Histogram of the percentage of individuals belonging to different disease score classes in 10 populations of *Zingiber zerumbet***.

With respect to the disease score, the ten *Z. zerumbet* populations could be separated into two groups, one consisting of three south Kerala populations sampled from the forest ranges (ZzKLR, ZzAPR, and ZzKLZ) and the other consisting of seven populations sampled from revenue lands (ZzKTK, ZzPNR, ZzPVD, ZzKLD, ZzPZR, ZzCLT, and ZzKNR). Of the 163 plants screened in total from the three south Kerala populations, 85 plants (52%) were wilted within 25 dpi (score 9), whereas in the remaining 78 plants, score 7, 5 and 3 were recorded in 32, 18 and 15 plants respectively. Altogether 13 plants were immune to the disease (Supplementary Table S2c). Conversely, in the seven populations collected from revenue lands, 185 plants (85%) out of the 213 plants screened in total were immune to the disease. In the remaining 28 plants, the disease score ranged from 1 (14 plants) to score 7 (three plants) with none yielding score 9 (**Figure 2**; Supplementary Table S2c).

### Nature and Extent of AFLP Diversity in *Zingiber* spp.

The AFLP error rate obtained in ginger (0.11%), *Z. neesanum* (0.82%), *Z. nimmonii* (0.78%), and *Z. zerumbet* (0.26%) was negligible as compared to the AFLP error rate reported earlier in other species, such as *Juniperus communis* (12.6%, Vanden-Broeck et al., 2011) and *Campanula sabatia* (1.56%, Nicoletti et al., 2012). Population genetic parameters computed in the four *Zingiber* spp. based on AFLP data are given in **Table 2** and the total and the polymorphic fragments produced by each primer combination in a species are given in Supplementary Table S4. ANOVA showed very high statistical significance (*p*= 0.0000019) in *h* between populations.

**Table 2:** Genetic diversity characteristics of *Zingiber* spp. based on amplified fragment length polymorphism data.

Cultivar/population^∗^	PPB	*h*	*I*	Ar	Pa
***Z. officinale***					
Mahima (3)	0	0	0	1 ± 0	0
Varada (3)	0.49	0.0024 ± 0.0343	0.0034 ± 0.0478	1.0042 ± 0.0042	0.0042 ± 0.0042
Rejatha (3)	0.49	0.0015 ± 0.0210	0.0023 ± 0.0335	1.0042 ± 0.0042	0
Bhaisey (3)	0.49	0.0015 ± 0.0210	0.0023 ± 0.0335	1.0042 ± 0.0042	0
Himgiri (3)	0.49	0.0015 ± 0.0211	0.0023 ± 0.0336	1.0042 ± 0.0042	0
Nadia (3)	0	0	0	1 ± 0	0
Suprabha (3)	0.99	0.0039 ± 0.0401	0.0057 ± 0.0582	1.0084 ± 0.0059	0
Suruchi (3)	0.99	0.003 ± 0.0297	0.0047 ± 0.0472	1.0084 ± 0.0059	0
Suravi (3)	1.48	0.0054 ± 0.0452	0.0081 ± 0.0669	1.0126 ± 0.0072	0
V3S1-8 (3)	0	0	0	1 ± 0	0
Athira (3)	0.49	0.0024 ± 0.0343	0.0034 ± 0.0478	1.0042 ± 0.0042	0
Karthika (3)	0.49	0.0024 ± 0.0343	0.0034 ± 0.0478	1.0042 ± 0.0042	0
Maran (3)	0	0	0	1 ± 0	0
Species level	2.4	0.009 ± 0.0619	0.0133 ± 0.0888		
***Z. neesanum***					
ZnTRL (10)	19.37	0.0696 ± 0.1540	0.1035 ± 0.2230	1.1384 ± 0.0165	0.3385 ± 0.0252
ZnWGN (10)	17.78	0.0648 ± 0.1491	0.0964 ± 0.2164	1.1311 ± 0.0163	0.0455 ± 0.0096
ZnPMD (10)	18.41	0.0686 ± 0.1557	0.1010 ± 0.2242	1.1351 ± 0.0166	0.0360 ± 0.0088
Species level	60.95	0.2400 ± 0.2077	0.3519 ± 0.2965		
***Z. nimmonii***					
ZcKNR (10)	15.89	0.0605 ± 0.1477	0.0890 ± 0.2133	1.1147 ± 0.0153	0.0274 ± 0.0075
ZcCU (10)	21.18	0.0837 ± 0.1713	0.1219 ± 0.2451	1.1606 ± 0.0178	0.0096 ± 0.0042
ZcTRL (10)	32.09	0.1318 ± 0.2013	0.1908 ± 0.2871	1.2466 ± 0.0207	0.0199 ± 0.0056
ZcVRR (10)	31.78	0.1210 ± 0.1860	0.1790 ± 0.2704	1.2527 ± 0.0212	0.0285 ± 0.0074
ZcPVD (10)	24.3	0.0914 ± 0.1724	0.1348 ± 0.2492	1.1937 ± 0.0197	0.0401 ± 0.0093
Species level	48.91	0.16380 ± 0.2007	0.2514 ± 0.2861		
***Z. zerumbet***					
ZzKNR (10)	0.3	0.0013 ± 0.0238	0.0019 ± 0.0343	1.0022 ± 0.0022	0
ZzCLT (10)	0.3	0.0003 ± 0.0054	0.0006 ± 0.0111	1.0022 ± 0.0022	0
ZzPZR (10)	0	0	0	1 ± 0	0
ZzKLD (10)	0.3	0.0003 ± 0.0055	0.0006 ± 0.0112	1.0022 ± 0.0022	0
ZzPVD (10)	0.91	0.0038 ± 0.0393	0.0055 ± 0.0574	1.0086 ± 0.0049	0
ZzKTK (10)	61.33	0.244 ± 0.2111	0.3564 ± 0.2996	1.548 ± 0.0244	0.2210 ± 0.0046
ZzPNR (10)	65.26	0.2497 ± 0.2058	0.3673 ± 0.2911	1.5783 ± 0.0238	0.0065 ± 0.0030
ZzKLZ (20)	80.97	0.3047 ± 0.1839	0.4493 ± 0.2549	1.6604 ± 0.0199	0.022 ± 0.0055
ZzAPR (20)	79.76	0.2999 ± 0.1896	0.4412 ± 0.2630	1.6520 ± 0.0201	0.0153 ± 0.0038
ZzKLR (20)	77.34	0.2787 ± 0.1954	0.4122 ± 0.2714	1.5966 ± 0.0203	0.0231 ± 0.0052
Species level	94.26	0.3607 ± 0.1530	0.5288 ± 0.1992		

*PPB, percentage polymorphic bands; h, Nei’s genetic diversity; I, Shannon information Index; Ar, Allele richness; Pa, Private allele richness. ^∗^Number of individuals used per cultivars or populations is in parenthesis.*

All the genetic diversity parameters were extremely low or nil in the 13 cultivars of the obligately asexual ginger. The 13 cultivars yielded altogether only five polymorphic fragments (PPB = 2.4), out of the 203 fragments produced in total by five AFLP primer combinations (**Table 2**; Supplementary Table S4). The five polymorphic fragments were shared between nine cultivars and no Pa were detected in cultivars (**Table 2**). The primer combinations E-ACT × M-CTA and E-AGC × M-CTC produced three and two polymorphic fragments, respectively, whereas the remaining three combinations produced only monomorphic fragments (Supplementary Table S4; Supplementary Figures S1a,b). No polymorphism was found within cultivars.

The diversity analysis yielded relatively high values for all the diversity parameters in the two seed setting species, *Z. neesanum* and *Z. nimmonii*. In *Z. neesanum* and *Z. nimmonii*, 192 out of 315 fragments (PPB = 60.95) and 157 out of 321 fragments (PPB = 48.91) were polymorphic, respectively (**Table 2**; Supplementary Table S4; Supplementary Figures S2 and S3). In *Z. neesanum*, at the population level, ZnTRL population yielded the highest PPB of 19.37%, followed by 18.41% in ZnPMD and 17.78% in ZnWGN. In *Z. nimmonii*, the PPB ranged between 15.89 (ZcKNR) and 32.09 (ZcTRL) (**Table 2**). Corresponding with the PPB data, the *h, I* and Ar values were also relatively high at the species and population levels (**Table 2**) in both *Z. neesanum* and *Z. nimmonii*. The distribution of Pa was low in *Z. neesanum* and *Z. nimmonii* populations except in the *Z. neesanum* population ZnTRL.

*Zingiber zerumbet* yielded the highest level of polymorphism among the four *Zingiber* spp. analyzed with 312 fragments being polymorphic (PPB = 94.26) out of the 331 fragments produced by six primer combinations (**Table 2**; Supplementary Table S4). However, the level of genetic diversity within population differed markedly between the populations of *Z. zerumbet*. At the population level, five of the seven non-seed setting *Z. zerumbet* populations from revenue land yielded practically negligible PPB: ‘0’ (no polymorphic fragment) in ZzPZR, 0.3 (one polymorphic fragment) each in ZzKNR, ZzCLT and ZzKLD and 0.9 (three polymorphic fragments) in ZzPVD (**Table 2**; Supplementary Figure S4a). The *h, I* and Ar values were also correspondingly low in these populations and none of the populations produced Pa (**Table 2**). In opposite to this, the remaining five populations, including the two non-seed-setting populations from revenue land (ZzKTK and ZzPNR) and all of the three seed-setting populations from forest ranges (ZzKLZ, ZzKLR, and ZzAPR), were highly diverse (Supplementary Figure S4b) and yielded very high values for PPB, *h, I* and Ar (**Table 2**). ADZE analysis produced low Pa for these populations, except in ZzKTK (**Table 2**). Besides, the inter-population comparison of AFLP profiles depicted the occurrence of two distinct multi-locus genotypes within *Z. zerumbet* populations; one shared between ZzPVD and ZzKLD and the other between ZzPZR, ZzCLT, and ZzKNR (Supplementary Figure S4a). Only four fragments were polymorphic between ZzPVD and ZzKLD and 14 fragments between ZzPZR, ZzCLT and ZzKNR.

### Population Genetic Structure in *Zingiber* spp.

**FIGURE 3:**
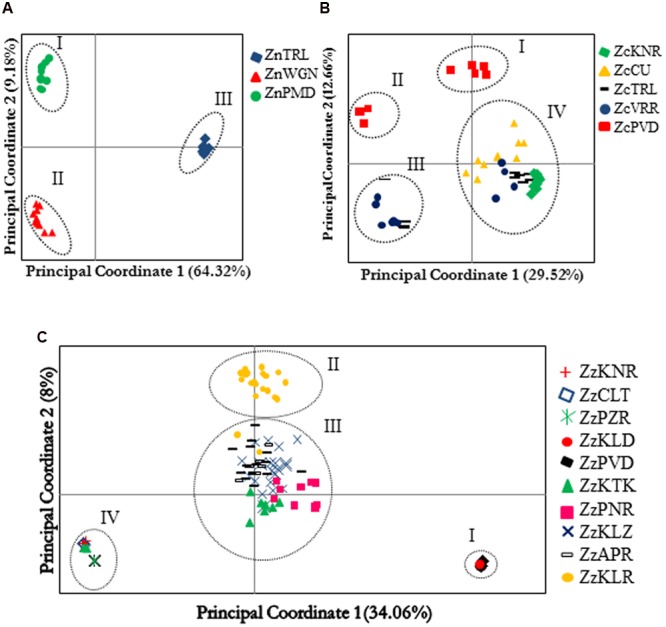
**Principal component analysis of the individuals belonging to different populations of *Zingiber neesanum* (A)**, *Zingiber nimmonii* **(B)**, and *Z. zerumbet* **(C)** based on amplified fragment length polymorphism data. Clusters identified in each species are numbered.

No population genetic analysis was performed on the AFLP data generated in ginger, as the detected diversity was scarce in this species. The first three principal coordinates obtained by the PCoA of Nei’s genetic distances computed from AFLP data explained 76.12%, 51.15%, and 46.55% of the total variations in *Z. neesanum, Z. nimmonii*, and *Z. zerumbet* respectively. Scatter plot of the first two principal coordinates (PCos) was generated in each species (**Figures 3A–C**) and examined the pattern of population sub-division within a species together with the results produced by Bayesian algorithm STRUCTURE (Supplementary Figures S5a–f).

Principle coordinate analysis clearly separated the three *Z. neesanum* populations into distinct groups (**Figure 3A**). Population sub-division produced by STRUCTURE analysis (Supplementary Figures S5a,b) was similar to the grouping pattern yielded by PCoA (**Figure 3A**). In *Z. nimmonii*, the PCoA depicted four distinct groups (**Figure 3B**). The ZcPVD individuals were separated into two distinct groups: group I and group II. A few individuals each from ZcTRL and ZcVRR entered into group III while the remaining individuals from these populations were nested with individuals from ZcKNR and ZcCU populations and formed the group IV. The four subpopulations identified by Evanno’s Δ*K* statistics at *K*= 4 (Supplementary Figures S5c,d) corresponded with the results of PCoA (**Figure 3B**). Bayesian algorithm detected inter-populational genetic admixing within *Z. nimmonii* (Supplementary Figure S5d).

Principle coordinate analysis of AFLP data separated the 130 *Z. zerumbet* individuals into four distinct groups (**Figure 3C**). The two non-seed-setting populations, ZzPVD and ZzKLD, which shared one of the two distinct multi-locus genotypes, together produced group I while the other three non-seed-setting populations ZzPZR, ZzCLT, and ZzKNR, which shared the other multi-locus genotype, together with a few individuals from ZzKTK produced group IV. Most of the individuals belonging to the seed-setting population ZzKLR produced a distinct group (group II). Group III was heterogeneous and consisted of individuals belonging to the two seed-setting populations ZzKLZ and ZzAPR, all individuals of non-seed setting population ZzPNR and most of the individuals from non-seed setting population ZzKTK. The pattern of population sub-division revealed by STRUCTURE mostly corresponded with the results of PCoA (**Figure 3C**) at *K* = 4 (Supplementary Figures S5e,f). STRUCTURE detected genetic admixing in *Z. zerumbet*, especially between the individuals entered into the PCoA group III. This admixing was evident in UPGMA dendrogram also, which supported the grouping patterns produced by PCoA group I, group II, and group IV in *Z. zerumbet*, but resolved the PCoA group III into a finer scale (Supplementary Figure S6). The ZzPNR population, despite its geographic proximity to ZzKTK and ZzKLZ (Supplementary Table S3c), clustered closer to the cluster consisted of ZzPVD and ZzKLD, which constituted the PCoA group I. Likewise, three ZzKTK individuals entered into the cluster which consisted of PCoA group IV populations, ZzPZR, ZzCLT, and ZzKNR, while the remaining seven individuals formed a distinct cluster (Supplementary Figure S6).

Analysis of molecular variance revealed significant (*p*< 0.05) between population partitioning of total genetic variability in *Z. neesanum* (*F*_st_ = 0.78), *Z. nimmonii* (*F*_st_ = 0.37), and *Z. zerumbet* (*F*_st_ = 0.5) (Supplementary Tables S5a–c). The pair-wise *F*_st_ varied drastically in *Z. zerumbet*. The pair-wise *F*_st_ values between the populations within the PCoA group I and the IV were ≤0.075 whereas the value was ≥0.99 between group I and IV populations (Supplementary Table S5c). Genetic differentiation between the two non-setting populations ZzKTK and ZzPNR and the two nearby (Supplementary Table S3c) seed setting populations ZzKLZ and ZzAPR was relatively low (*F*_st_ ≤ 0.2; *p <*0.05) (Supplementary Table S5c). Gene flow was low in *Z. neesanum* (Nm = 0.1964) whereas it was relatively moderate and high in Z. *zerumbet* (Nm = 0.32) and *Z. nimmonii* (Nm = 0.6921), respectively.

## Discussion

### Reproductive Strategies in *Zingiber* spp.

The level of seed set in natural habitat as well as the percentage germination of seeds determined in the laboratory (**Table 1**) depicted sexual reproduction in *Z. neesanum* and *Z. nimmonii*, as reported in other plant species (Tiébré et al., 2007), and a mixed reproductive behavior in *Z. zerumbet*, consisting of sexually reproducing populations in forest ranges and clonal populations in revenue land. The mixed reproductive strategy has been reported earlier in aquatic plant *Decodon verticillatus* (Eckert et al., 1999). Thus, the three wild *Zingiber* spp. chosen in the study together with the obligately asexual cultivated congener, ginger constitute an ideal system to dissect how the reproductive strategy influences other biological functions of a plant. Hereafter, we refer the reproductive strategy observed in *Z. zerumbet* as “hemiclonal”. Because the terminologies such as “mixed mating” or “mixed clonal/sexual” that are commonly used in literature to denote this kind of mating system (McKey et al., 2010) may be confusing as these terms are often used to indicate other breeding systems also (Goodwillie et al., 2005). In addition, as discussed later, the clonal and sexual populations in *Z. zerumbet* are sub-divided spatially between the ecologically distinct revenue lands and forest ranges.

### Population Genetic Characteristics *vis-à-vis* Breeding Strategy in *Zingiber* spp.

Consistent with the results of other studies reported earlier (Pfeiffer et al., 2012; Godfrey and Johnson, 2014), high genetic diversity was recorded in the sexually reproducing *Z. neesanum* and Z. *nimmonii* populations and the three south Kerala populations of *Z. zerumbet* from forest land and narrow genetic base in obligately asexual ginger and five of the seven clonal populations of *Z. zerumbet* from revenue land (**Table 2**). The level of genetic diversity in two clonal populations of *Z. zerumbet*, ZzKTK and ZzPNR, represented an exception, which, as discussed later, can be addressed in the light of available literature.

The population genetic parameters obtained in the study permit a discussion about certain characteristics of the population genetic structure of *Zingiber* spp. that have a potential bearing on population fitness. Despite analyzing cultivars sampled from different parts of India, the genetic diversity in ginger was practically nil (**Table 2**; Supplementary Table S4; Supplementary Figures S1a,b). Certain authors have reported the occurrence of diversity in random amplified polymorphic DNA (RAPD) markers in Indian cultivars of ginger (Sajeev et al., 2011) however, the agarose gel pictures of PCR products depicting the patterns of variability between the cultivars used in their study are not provided in such articles for confirmation. Molecular fingerprinting studies indicate that the clonal populations may be a product of recent evolutionary events occurred in sexual populations (Vrijenhoek, 1998; Tucker et al., 2013), and, usually, multiple such events may occur in a species at different occasions, resulting in the emergence of multiple clonal lineages (Vrijenhoek, 1998). Conversely, AFLP results illustrates that in India ginger is likely represented by a single clone (monoclonal), may an “ecological generalist” (Vrijenhoek, 1998). It may be possible that ginger may have had originated only once in the evolutionary past. Monoclonal species are extremely rare in literature. Based on AFLP studies, Pfeiffer et al. (2012) have earlier reported monoclonality in *G. spathacea* (Liliaceae).

Endemic species are generally considered to be genetically narrow (Gitzendanner and Soltis, 2000). However, in contrast to this general assumption, the South Indian endemic *Z. neesanum* and Z. *nimmonii* (Sabu, 2003) yielded high genetic diversity (**Table 2**), as reported before in certain other endemic species such as *C. sabatia* (Nicoletti et al., 2012). The significant (*p*< 0.05) population differentiation observed in *Z. neesanum* (*F*_st_ = 0.70) may be a corollary of very low gene flow (Nm = 0.1964) observed in this species, because the populations of a species with low gene flow are tended to differentiate locally (Ellstrand, 2014). In *Z. nimmonii*, despite the relatively higher gene flow (Nm = 0.6921), AMOVA revealed significant (*p*< 0.05) local differentiation (*F*_st_ = 0.37). Population differentiation amid gene flow has been reported earlier in *Eperua falcata* (Fabaceae) and is hypothesized due to molecular divergence associated with local adaptation under heterogeneous environmental conditions (Audigeos et al., 2013).

Population genetic structure of *Z. zerumbet* is in fact a product of its hemiclonality. Sexual populations of *Z. zerumbet* (ZzKLR, ZzAPR, and ZzKLZ) were genetically diverse while clonal populations (ZzPVD, ZzKLD, ZzPZR, ZzCLT, and ZzKNR), except the ZzKTK and ZzPNR populations, were genetically narrow (**Table 2**). The ZzKTK and ZzPNR populations that sparingly produced seeds in experimental conditions may be rarely producing seeds in natural habitats also. The relatively low genetic differentiation (*F*_st_ ≤ 0.02; *p*< 0.05) (Supplementary Table S5c) observed between these populations and the nearby (Supplementary Table S3c) seed setting populations ZzKLZ and ZzAPR further indicates a possible occurrence of gene flow between them. Thus the high genetic diversity recorded in ZzKTK and ZzPNR populations support the theoretical models, which predict that the populations with “little sex” can be as diverse as sexual populations (Green and Noakes, 1995; D’Souza and Michiels, 2010). The ZzPVD and ZzKLD populations shared one of the two clonal genotypes identified in the study whereas the ZzPZR, ZzCLT, and ZzKNR populations shared the other genotype. Multiple clonal lineages have been reported in other clonal species also (Vrijenhoek, 1998) and the data suggest occasional emergence of clonality in genetically distinct sexual populations of *Z. zerumbet*.

### Pathogen Responsiveness *vis-à-vis* Reproduction Strategy and Population Genetic Characteristics in *Zingiber* spp.

Although both were typically clonal, the ginger was highly susceptible to *P. aphanidermatum*, while the *Z. zerumbet* populations from revenue land were immune to it. Taken together, the results demonstrate that the continuous asexual reproduction lead populations into low genetic diversity levels (Rice, 2002; Pfeiffer et al., 2012), but pathogen susceptibility cannot be assumed *ipso facto* in such populations as generally believed (Keesing et al., 2010). This indicates that the mechanics of the eco-evolutionary feedback that trigger clonality in a population may be the primary factor determining the nature of genetical effect that clonality can contribute to a population.

It can be presumed that the clonality may have been triggered in ginger following a recent genomic disturbance, as reported in other systems, in order to counteract the adversities of sterility caused by such disturbances on taxon persistence (Vrijenhoek, 1998; Janko et al., 2008; Tucker et al., 2013). By reading together the obligate asexual propagation, exceptionally narrow genetic diversity and the high susceptibility to *P. aphanidermatum*, we can presume that Muller’s ratchet (Rice, 2002) operates in ginger. Thus host-pathogen co-evolution is ineffective in ginger (Rice, 2002; Morran et al., 2011), rendering ginger highly susceptible not only to soft-rot disease but also to many other diseases such as bacterial wilt, *Fusarium* yellows and *Phyllotica* leaf spot caused by *Ralstonia solanacearum, Fusarium oxysporum* f. sp. *zingiberi* and *Phyllosticta zingiberi*, respectively (Dake, 1995; Le et al., 2014). A few instances of Muller’s ratchet have been reported in animals and microorganisms (Kaiser and Charlesworth, 2010; Jaramillo et al., 2013). Somatic mutations that are known to generate genetic variability in asexuals (Infante et al., 2003) seem not fastened in ginger, suggesting that the background selection (Rice, 2002) may be operating strongly in ginger.

On the other hand, clonality must be an outcome of ecological adaptation in *Z. zerumbet*. The results of computer simulations performed by Lively et al. (1994), show that the repeated mutations in sexual populations to parthenogenesis can lead to the accumulation of clones with different level of pathogen resistance and the descendants of certain adaptive mutants may replace the ancestral sexual populations. Studies of King et al. (2011) in snails supported the computer simulations of Lively et al. (1994) and showed further that the parasitic infection not only increased the diversity of sexual individuals in tune with theoretical expectations (Morran et al., 2011), but also promoted the emergence of different parthenogenetic subpopulations from sexual populations by mutations (King et al., 2011). Taken together, we can presume that the *P*. *aphanidermatum* resistant *Z. zerumbet* populations in revenue lands are the descendants of mutants emerged in sexual populations and expanded clonally. A single recessive mutation is capable of triggering clonality in sexual populations (Eckert et al., 1999). Despite being geographically closer to the three *Pythium* susceptible sexual populations in southern Kerala (ZzKLR, ZzAPR, and ZzKLZ) (Supplementary Table S3c), the resistant non seed-setting ZzKTK and ZzPNR populations were genetically closer to the geographically distant resistant clonal populations from northern Kerala (ZzPVD, ZzKLD, ZzPZR, ZzCLT, and ZzKNR) (Supplementary Figure S6). The data support the argument of Campbell (2015) that the defense and reproduction may have reciprocal and coevolutionary effects on each other. A sexual organism with genetical potential for clonal multiplication (hemiclonality) can tolerate mutations (Ram and Hadany, 2012) and, thus, are capable of harvesting the adaptive benefits of both sexuality and clonality (Ram and Hadany, 2012; Hojsgaard and Horandl, 2015); thereby broadening its genetic base, which in turn indicate a possibility that, for adaptive fitness, diversity matters but not necessarily the sex (D’Souza and Michiels, 2010; Seidl and Thomma, 2014). Thus, hemiclonality renders an organism the capability to swing between sexual and clonal reproduction as per the ecological demands, thereby facilitate its long range dispersal by clonal expansion unaffected by reduced sexual fitness caused by mate limitations (Van Drunen et al., 2015). Incidentally, *Z. zerumbet* is distributed abundantly in south and Southeast Asian countries (Sabu, 2003). Adaptive fitness of similar reproductive strategies, such as cyclical parthenogenesis, has long been recognized in many organisms (D’Souza and Michiels, 2010).

Yet, the ecological factors prevailing in forest understory and revenue land may have had complemented the trade-offs between sexual and clonal recruits in *Z. zerumbet* populations. In fragmented populations, such as *Z. zerumbet* populations in revenue land, clonality may persist due to limited reproductive success (Binks et al., 2015), local extinction of pathogen due to their slow dispersal between isolated patches (Carlsson-Granér and Thrall, 2002) and poor colonization of *Pythium* spp. on roots exposed to sunlight (Hayden et al., 2013). On the contrary, in the large inter-connected understory populations of *Z. zerumbet* from southern Kerala, the pathogen may persists by their frequent dispersal between host patches and exerts continuous stress on hosts (Carlsson-Granér and Thrall, 2002), prompting the host to resort sexual recruits for want of new variants (Morran et al., 2011). In addition, the cooler and dense environment in the forest promotes *Pythium* disease (Hayden et al., 2013). Thus, due to different ecological reasons sexual recruits persists in south Kerala populations from forest ranges, resulting in continuous segregation of resistance trait emerges in these populations and diluting local adaptation (Cremieux et al., 2010).

Contrary to the expectations of Red Queen hypothesis (Clay and Kover, 1996; Morran et al., 2011), the genetically diverse *Z. neesanum* and *Z. nimmonii* populations were highly susceptible to *P. aphanidermatum*. The data suggest that genetic variability can be generated by virtue of sexual reproduction, but resistance specificities do not necessarily emerge concomitantly. Further, the data suggest that, by default the genetic diversity do not buffer the host against biotic stress (Hendry, 2013) and we still have a long way to go to set “diversity thresholds” (King and Lively, 2012). The lack of resistance in *Z. neesanum* and *Z. nimmonii* populations and the complete destruction of the host populations by *P. aphanidermatum* raise a possibility that the *Z. neesanum* and *Z. nimmonii* populations and *P. aphanidermatum* were not cohabited in the past for sufficiently longer period for the co-evolutionary trajectories to shape resistance specificities in the host and virulence characteristics in the pathogen (Antonovics et al., 2012). Another possibility is that, resistance against the necrotrophic pathogen is quantitative (Rowe and Kliebenstein, 2008) and as opined by Clay and Kover (1996), quantitative resistance may not evolve frequently by frequency dependent selection (FDS) by evolutionary arms race as postulated in Red Queen hypothesis.

## Conclusion

The study deciphered a key role for breeding behavior in deciding the survival fitness and population expansion. The obligate asexuality drives the population into genetic shallowness, at the same time; genetic diversity, generated consequential to sexuality, alone does not guarantee population fitness. The hemiclonality should be viewed as an evolutionary destination with a positive effect on species continuum. Species with clonal traits are increasingly being identified in different kinds of living organisms (Neaves and Baumann, 2011) and clonal biology has recently gained a great momentum in several laboratories considering the adaptive potential of clonal organisms in extreme environments (Johnson et al., 2010; Klimesova and Pysek, 2011; Tibayrenc et al., 2015). It may be possible that, when genetic recombination becomes at a premium due to the genetic constraints imparted by ecological and climatic factors such as habitat fragmentation or global warming, plants may trigger asexual methods in order to carefully preserve genotypes with adaptive fitness. Presumably, species with potential ability to propagate clonally may have a fair chance to survive ecological undulations.

## Author Contributions

GET, KG and LA performed plant collection and maintenance, inoculation experiments, genotyping using AFLP and analysis of data. SM conducted field survey and identified populations. GT designed the work, evaluated the data and prepared the manuscript.

## Conflict of Interest Statement

The authors declare that the research was conducted in the absence of any commercial or financial relationships that could be construed as a potential conflict of interest.
