# Supplementary material for: Analyses between Reproductive Behavior, Genetic Diversity and Pythium Responsiveness in Zingiber spp. Reveal an Adaptive Significance for Hemiclonality
Source: Front Plant Sci. 2016 Dec 20;7:1913. doi: 10.3389/fpls.2016.01913 (PMC5167741; doi:10.3389/fpls.2016.01913)
Supplement: Supplementary file 1 [file Table_1.DOC]

**Supplementary Table**

**Table S1** List of individuals of the 13cultivars of ginger (*Z. officinale*) used in the study together with their disease score

| Sl. No. | Cultivar (Individual name in parenthesis) | Code No. | Origin/ Source** | Disease Score |
| --- | --- | --- | --- | --- |
| 1 | Mahima [5 (1)]*¥ | ZoMHM01 | IISR | 9 |
| 2 | Mahima [9 (1)]* ¥ | ZoMHM02 | IISR | 9 |
| 3 | Mahima [9 (2)]* ¥ | ZoMHM03 | IISR | 9 |
| 4 | Mahima [16 (1)] | ZoMHM04 | IISR | 9 |
| 5 | Mahima [16 (2)] | ZoMHM05 | IISR | 9 |
| 6 | Varada [2 (1)]* ¥ | ZoVAR01 | IISR | 9 |
| 7 | Varada [2 (2)]* ¥ | ZoVAR02 | IISR | 9 |
| 8 | Varada [4 (1)] | ZoVAR03 | IISR | 9 |
| 9 | Varada [4 (2)] | ZoVAR04 | IISR | 9 |
| 10 | Varada [15 (1)] | ZoVAR05 | IISR | 9 |
| 11 | Varada [15 (2)]* ¥ | ZoVAR06 | IISR | 9 |
| 12 | Rejatha [9 (1)] | ZoREJ01 | IISR | 9 |
| 13 | Rejatha [9 (2)] | ZoREJ02 | IISR | 9 |
| 14 | Rejatha [15 (1)]* ¥ | ZoREJ03 | IISR | 9 |
| 15 | Rejatha [15 (2)]* ¥ | ZoREJ04 | IISR | 9 |
| 16 | Rejatha [22 (1)]* ¥ | ZoREJ05 | IISR | 9 |
| 17 | Rejatha [22 (2)] | ZoREJ06 | IISR | 9 |
| 18 | Bhaisey[2 (2)]* ¥ | ZoBYS01 | CAU | 9 |
| 19 | Bhaisey[2 (1)]* ¥ | ZoBYS02 | CAU | 9 |
| 20 | Bhaisey[3 (1)]* ¥ | ZoBYS03 | CAU | 9 |
| 21 | Himgiri [9 (1)]* ¥ | ZoHMG01 | YSPUH | 9 |
| 22 | Himgiri[9 (2)] | ZoHMG02 | YSPUH | 9 |
| 23 | Himgiri[12 (1)]* ¥ | ZoHMG03 | YSPUH | 9 |
| 24 | Himgiri[12 (2)]* ¥ | ZoHMG04 | YSPUH | 9 |
| 25 | Nadia[2 (1)] | ZoNAD01 | CAU | 9 |
| 26 | Nadia[3 (1)]* ¥ | ZoNAD02 | CAU | 9 |
| 27 | Nadia[3 (2)]* ¥ | ZoNAD03 | CAU | 9 |
| 28 | Nadia[4 (1)]* ¥ | ZoNAD04 | CAU | 9 |
| 29 | Suprabha [2029 (1)]* | ZoSUP01 | OUAT | 9 |
| 30 | Suprabha [2029 (2)]* | ZoSUP02 | OUAT | 9 |
| 31 | Suprabha [2029 (3)]* | ZoSUP03 | OUAT | 9 |
| 32 | Suruchi [2028 (1)]* | ZoSUR01 | OUAT | 9 |
| 33 | Suruchi [2028 (3)]* | ZoSUR02 | OUAT | 9 |
| 34 | Suruchi [2028 (4)]* | ZoSUR03 | OUAT | 9 |
| 35 | Suruchi [2028 (2)] | ZoSUR04 | OUAT | 9 |
| 36 | Suravi [2030 (1)]* | ZoSRV01 | OUAT | 9 |
| 37 | Suravi [2030 (2)]* | ZoSRV02 | OUAT | 9 |
| 38 | Suravi [2030 (3)]* | ZoSRV03 | OUAT | 9 |
| 39 | V3S1-8 [2032 (1)]* | ZoVVS01 | OUAT | 9 |
| 40 | V3S1-8 [2032 (2)]* | ZoVVS02 | OUAT | 9 |
| 41 | V3S1-8 [2032 (3)]* | ZoVVS03 | OUAT | 9 |
| 42 | Athira [2025 (10)]* | ZoATH01 | KAU | 9 |
| 43 | Athira [2025 (7)]* | ZoATH02 | KAU | 9 |
| 44 | Athira [2025 (8)]* | ZoATH03 | KAU | 9 |
| 45 | Athira [2025 (1)] | ZoATH04 | KAU | 9 |
| 46 | Karthika [2024 (9)]* | ZoKAR01 | KAU | 9 |
| 47 | Karthika [2024 (1)]* | ZoKAR02 | KAU | 9 |
| 48 | Karthika [2024 (2)]* | ZoKAR03 | KAU | 9 |
| 49 | Karthika [2024 (3)] | ZoKAR04 | KAU | 9 |
| 50 | Karthika [2024 (5)] | ZoKAR05 | KAU | 9 |
| 51 | Maran [2026 (1)] | ZoMAR01 | Sulthan Bathery, Kerala | 9 |
| 52 | Maran [2026 (2)] | ZoMAR02 | Sulthan Bathery, Kerala | 9 |
| 53 | Maran [2026 (24)]* | ZoMAR03 | Sulthan Bathery, Kerala | 9 |
| 54 | Maran [2026 (8)]* | ZoMAR04 | Sulthan Bathery, Kerala | 9 |
| 55 | Maran [2026 (12)]* | ZoMAR05 | Sulthan Bathery, Kerala | 9 |
| 56 | Maran [2026 (4)] | ZoMAR06 | Sulthan Bathery, Kerala | 9 |
| * Samples included in AFLP analysis  ** IISR: Indian Institute of Spices Research, Calicut, Kerala  CAU: Central Agricultural University, Imphal, Manipur  YSPUH: Dr. Y. S. Parmer University of Horticulture and Forestry, Solan, Himachal Pradeesh  OUAT: Orissa University of Agriculture and Technology, Bhubaneswar, Odisha  KAU: Kerala Agricultural University, Thrissur, Kerala  ¥ Individuals used in initial primer screening and for the estimation for AFLP error rate | | | | |

**Table S2** List of accessions belonging to different populations of the three wild *Zingiber* spp. used in the study together with their disease score

| **S2a** *Z. neesanum* | | | | |
| --- | --- | --- | --- | --- |
| Sl. No. | Accession Number | Code No. | Disease Score | |
|  |  |  |  | |
| I Population: ZnTRL (Collecting site Thirunelli) | | | | |
| 1 | Zn TRNL 1(1) | ZnTRL01 | 9 | |
| 2 | Zn TRNL 1(2) | ZnTRL02 | 9 | |
| 3 | Zn TRNL 1(3) | ZnTRL03 | 9 | |
| 4 | Zn TRNL 1(4) | ZnTRL04 | 9 | |
| 5 | Zn TRNL 2(1)* ¥ | ZnTRL05 | 9 | |
| 6 | Zn TRNL 2(2)* | ZnTRL06 | 9 | |
| 7 | Zn TRNL 2(3)* | ZnTRL07 | 9 | |
| 8 | Zn TRNL 2(4)* | ZnTRL08 | 9 | |
| 9 | Zn TRNL 3(1)* | ZnTRL09 | 9 | |
| 10 | Zn TRNL 3(2) | ZnTRL10 | 9 | |
| 11 | Zn TRNL 3(3) | ZnTRL11 | 9 | |
| 12 | Zn TRNL 3(4) | ZnTRL12 | 9 | |
| 13 | Zn TRNL 4(1)* | ZnTRL13 | 9 | |
| 14 | Zn TRNL 4(2) | ZnTRL14 | 9 | |
| 15 | Zn TRNL 4(3) | ZnTRL15 | 9 | |
| 16 | Zn TRNL 4(4) | ZnTRL16 | 9 | |
| 17 | Zn TRNL 5(1)* | ZnTRL17 | 9 | |
| 18 | Zn TRNL 5(2) | ZnTRL18 | 9 | |
| 19 | Zn TRNL 5(3)* | ZnTRL19 | 9 | |
| 20 | Zn TRNL 5(4)* | ZnTRL20 | 9 | |
| 21 | Zn TRNL 8(1)^*#^ | ZnTRL21 |  | |
|  |  |  |  | |
| II Population: ZnWGN (Collecting site Wagamon) | | | | |
| 22 | Zn WGMN-8(1)* | ZnWGN01 | 5 | |
| 23 | Zn WGMN-5(1) | ZnWGN02 | 9 | |
| 24 | Zn WGMN-5(2) | ZnWGN03 | 5 | |
| 25 | Zn WGMN-5(3) | ZnWGN04 | 9 | |
| 26 | Zn WGMN-2(1)* | ZnWGN05 | 9 | |
| 27 | Zn WGMN-2(2)* | ZnWGN06 | 9 | |
| 28 | Zn WGMN-7(1) | ZnWGN07 | 3 | |
| 29 | Zn WGMN-7(2)* | ZnWGN08 | 3 | |
| 30 | Zn WGMN-6(1)* | ZnWGN09 | 9 | |
| 31 | Zn WGMN-6(2) | ZnWGN10 | 7 | |
| 32 | Zn WGMN-3(1) | ZnWGN11 | 9 | |
| 33 | Zn WGMN-3(2)* | ZnWGN12 | 9 | |
| 34 | Zn WGMN-3(3) | ZnWGN13 | 9 | |
| 35 | Zn WGMN-1(1)* | ZnWGN14 | 9 | |
| 36 | Zn WGMN-1(2) | ZnWGN15 | 9 | |
| 37 | Zn WGMN-1(3)* | ZnWGN16 | 9 | |
| 38 | Zn WGMN-4(1)* | ZnWGN17 | 9 | |
| 39 | Zn WGMN-4(2)* | ZnWGN18 | 9 | |
| 40 | Zn WGMN-4(3) | ZnWGN19 | 9 | |
| 41 | Zn VKLM-4(1) | ZnWGN20 | 9 | |
| 42 | Zn VKLM-4(2) | ZnWGN21 | 9 | |
| 43 | Zn VKLM-6(1) | ZnWGN22 | 9 | |
| 44 | Zn VKLM-6(2) | ZnWGN23 | 9 | |
| 45 | Zn VKLM-6(3) | ZnWGN24 | 9 | |
| 46 | Zn VKLM-3(1) | ZnWGN25 | 9 | |
| 47 | Zn VKLM-3(2) | ZnWGN26 | 9 | |
| 48 | Zn VKLM-3(3) | ZnWGN27 | 9 | |
| 49 | Zn VKLM-1(1) | ZnWGN28 | 9 | |
| 50 | Zn VKLM-1(2) | ZnWGN29 | 9 | |
| 51 | Zn VKLM-1(3) | ZnWGN30 | 9 | |
| 52 | Zn VKLM-1(4) | ZnWGN31 | 9 | |
| 53 | Zn VKLM-8(1) | ZnWGN32 | 9 | |
| 54 | Zn VKLM-8(2) | ZnWGN33 | 9 | |
| 55 | Zn VKLM-8(3) | ZnWGN34 | 9 | |
| 56 | Zn VKLM-7(1) | ZnWGN35 | 9 | |
| 57 | Zn VKLM-5(1) | ZnWGN36 | 9 | |
| 58 | Zn VKLM-5(2) | ZnWGN37 | 9 | |
| 59 | Zn VKLM-5(3) | ZnWGN38 | 9 | |
| 60 | Zn VKLM-2(1) | ZnWGN39 | 9 | |
| 61 | Zn VKLM-2(2) | ZnWGN40 | 9 | |
| 62 | Zn VKLM-2(3) | ZnWGN41 | 9 | |
| 63 | Zn VKLM-2(4) | ZnWGN42 | 9 | |
|  |  |  |  | |
| III Population: ZnPMD (Collecting site Ponmudi) | | | | |
| 64 | (8) Zn PNMD-4(1)* | ZnPMD01 | 9 | |
| 65 | (8) Zn PNMD-4(2) | ZnPMD02 | 9 | |
| 66 | (8) Zn PNMD-4(3) | ZnPMD03 | 9 | |
| 67 | (8) Zn PNMD-4(4) | ZnPMD04 | 9 | |
| 68 | (8) Zn PNMD-7(1) | ZnPMD05 | 9 | |
| 69 | (8) Zn PNMD-3(1)* | ZnPMD06 | 9 | |
| 70 | (8) Zn PNMD-3(2) | ZnPMD07 | 9 | |
| 71 | (8) Zn PNMD-3(3) | ZnPMD08 | 9 | |
| 72 | (8) Zn PNMD-9(1) | ZnPMD09 | 9 | |
| 73 | (8) Zn PNMD-1(1) | ZnPMD10 | 9 | |
| 74 | (8) Zn PNMD-1(2) | ZnPMD11 | 9 | |
| 75 | (8) Zn PNMD-8(1)* ¥ | ZnPMD12 | 9 | |
| 76 | (8) Zn PNMD-6(1)* | ZnPMD13 | 9 | |
| 77 | (8) Zn PNMD-6(2)* | ZnPMD14 | 9 | |
| 78 | (8) Zn PNMD-6(3)* | ZnPMD15 | 9 | |
| 79 | (8) Zn PNMD-5(1)* | ZnPMD16 | 9 | |
| 80 | (8) Zn PNMD-5(2)* | ZnPMD17 | 9 | |
| 81 | (8) Zn PNMD-5(3) | ZnPMD18 | 9 | |
| 82 | (8) Zn PNMD-5(4)* | ZnPMD19 | 9 | |
| 83 | (7) Zn PNMD-1(1) | ZnPMD20 | 9 | |
| 84 | Zn Ponmudi^*#^ | ZnPMD21 |  | |
|  |  |  |  | |
| **S2b** *Z*. *nimmonii* | | | | |
| Sl. No. | Accession Number | Code No. | Disease Score | |
|  |  |  |  | |
| I Population: ZcKNR (Collecting site Kannur) | | | | |
| 1 | Zc KNR 1(1) | ZcKNR01 | 9 | |
| 2 | Zc KNR 1(2) | ZcKNR02 | 9 | |
| 3 | Zc KNR 1(3) | ZcKNR03 | 9 | |
| 4 | Zc KNR 1(4) | ZcKNR04 | 9 | |
| 5 | Zc KNR 3(1) | ZcKNR05 | 9 | |
| 6 | Zc KNR 3(2)* | ZcKNR06 | 9 | |
| 7 | Zc KNR 3(3) | ZcKNR07 | 9 | |
| 8 | Zc KNR 3(4) | ZcKNR08 | 9 | |
| 9 | Zc KNR 4(1) | ZcKNR09 | 9 | |
| 10 | Zc KNR 4(2) | ZcKNR10 | 9 | |
| 11 | Zc KNR 4(3) | ZcKNR11 | 9 | |
| 12 | Zc KNR 4(4)* | ZcKNR12 | 3 | |
| 13 | Zc KNR 5(1) | ZcKNR13 | 7 | |
| 14 | Zc KNR 5(2) | ZcKNR14 | 7 | |
| 15 | Zc KNR 5(3)* | ZcKNR15 | 9 | |
| 16 | Zc KNR 5(4)* | ZcKNR16 | 9 | |
| 17 | Zc KNR 14(1) | ZcKNR17 | 9 | |
| 18 | Zc KNR 14(2) | ZcKNR18 | 9 | |
| 19 | Zc KNR 14(3) | ZcKNR19 | 9 | |
| 20 | Zc KNR 14(4) | ZcKNR20 | 7 | |
| 21 | Zc KNR 9(1)* | ZcKNR21 | 7 | |
| 22 | Zc KNR 9(2) | ZcKNR22 | 9 | |
| 23 | Zc KNR 9(3) | ZcKNR23 | 7 | |
| 24 | Zc KNR 9(4) | ZcKNR24 | 5 | |
| 25 | Zc KNR 26(1) | ZcKNR25 | 9 | |
| 26 | Zc KNR 26(2) | ZcKNR26 | 9 | |
| 27 | Zc KNR 26(3) | ZcKNR27 | 9 | |
| 28 | Zc KNR 26(4) | ZcKNR28 | 9 | |
| 29 | Zc KNR 11(1) | ZcKNR29 | 9 | |
| 30 | Zc KNR 11(2)* | ZcKNR30 | 9 | |
| 31 | Zc KNR 11(3) | ZcKNR31 | 9 | |
| 32 | Zc KNR 11(4)* | ZcKNR32 | 9 | |
| 33 | Zc KNR 12(1) | ZcKNR33 | 5 | |
| 34 | Zc KNR 12(2) | ZcKNR34 | 9 | |
| 35 | Zc KNR 12(3) | ZcKNR35 | 9 | |
| 36 | Zc KNR 12(4) | ZcKNR36 | 9 | |
| 37 | Zc KNR 19(1) | ZcKNR37 | 9 | |
| 38 | Zc KNR 19(2) | ZcKNR38 | 9 | |
| 39 | Zc KNR 19(3) | ZcKNR39 | 9 | |
| 40 | Zc KNR 19(4) | ZcKNR40 | 9 | |
| 41 | Zc KNR 20(1) | ZcKNR41 | 7 | |
| 42 | Zc KNR 20(2) | ZcKNR42 | 9 | |
| 43 | Zc KNR 20(3) | ZcKNR43 | 9 | |
| 44 | Zc KNR 20(4) | ZcKNR44 | 7 | |
| 45 | Zc KNR 21(1) | ZcKNR45 | 9 | |
| 46 | Zc KNR 21(2) | ZcKNR46 | 9 | |
| 47 | Zc KNR 21(3) | ZcKNR47 | 9 | |
| 48 | Zc KNR 21(4) | ZcKNR48 | 9 | |
| 49 | Zc KNR 22(1) | ZcKNR49 | 9 | |
| 50 | Zc KNR 22(2) | ZcKNR50 | 3 | |
| 51 | Zc KNR 22(3) | ZcKNR51 | 9 | |
| 52 | Zc KNR 22(4) | ZcKNR52 | 0 | |
| 53 | Zc KNR 23(1)* | ZcKNR53 | 9 | |
| 54 | Zc KNR 23(2)* | ZcKNR54 | 7 | |
| 55 | Zc KNR 23(3) | ZcKNR55 | 9 | |
| 56 | Zc KNR 23(4) | ZcKNR56 | 9 | |
| 57 | Zc KNR 24(1) | ZcKNR57 | 5 | |
| 58 | Zc KNR 24(2) | ZcKNR58 | 7 | |
| 59 | Zc KNR 24(3) | ZcKNR59 | 7 | |
| 60 | Zc KNR 24(4) | ZcKNR60 | 9 | |
| 61 | Zc KNR 10(1)^*#^ | ZcKNR61 |  | |
|  |  |  |  | |
| II Population: ZcTRL (Collecting site Thirunelli) | | | | |
| 62 | Zc TRNL 1(1) | ZcTRL01 | 9 | |
| 63 | Zc TRNL 1(2)* ¥ | ZcTRL02 | 9 | |
| 64 | Zc TRNL 1(3) | ZcTRL03 | 9 | |
| 65 | Zc TRNL 1(4)* | ZcTRL04 | 9 | |
| 66 | Zc TRNL 2(1) | ZcTRL05 | 9 | |
| 67 | Zc TRNL 2(2) | ZcTRL06 | 9 | |
| 68 | Zc TRNL 2(3) | ZcTRL07 | 9 | |
| 69 | Zc TRNL 2(4) | ZcTRL08 | 9 | |
| 70 | Zc TRNL 3(1) | ZcTRL09 | 9 | |
| 71 | Zc TRNL 3(2) | ZcTRL10 | 9 | |
| 72 | Zc TRNL 3(3)* | ZcTRL11 | 9 | |
| 73 | Zc TRNL 3(4) | ZcTRL12 | 9 | |
| 74 | Zc TRNL 4(1) | ZcTRL13 | 9 | |
| 75 | Zc TRNL 4(2) | ZcTRL14 | 9 | |
| 76 | Zc TRNL 4(3) | ZcTRL15 | 9 | |
| 77 | Zc TRNL 4(4) | ZcTRL16 | 9 | |
| 78 | Zc TRNL 5(1) | ZcTRL17 | 9 | |
| 79 | Zc TRNL 5(2) | ZcTRL18 | 9 | |
| 80 | Zc TRNL 5(3) | ZcTRL19 | 9 | |
| 81 | Zc TRNL 5(4) | ZcTRL20 | 9 | |
| 82 | Zc TRNL 6(1)* | ZcTRL21 | 9 | |
| 83 | Zc TRNL 6(2) | ZcTRL22 | 9 | |
| 84 | Zc TRNL 6(3) | ZcTRL23 | 9 | |
| 85 | Zc TRNL 6(4) | ZcTRL24 | 9 | |
| 86 | Zc TRNL 7(1)* | ZcTRL25 | 9 | |
| 87 | Zc TRNL 7(2) | ZcTRL26 | 9 | |
| 88 | Zc TRNL 7(3) | ZcTRL27 | 9 | |
| 89 | Zc TRNL 7(4) | ZcTRL28 | 9 | |
| 90 | Zc TRNL 8(1)* | ZcTRL29 | 5 | |
| 91 | Zc TRNL 8(2) | ZcTRL30 | 9 | |
| 92 | Zc TRNL 8(3) | ZcTRL31 | 9 | |
| 93 | Zc TRNL 8(4) | ZcTRL32 | 9 | |
| 94 | Zc TRNL 10(1) | ZcTRL33 | 9 | |
| 95 | Zc TRNL 10(2) | ZcTRL34 | 9 | |
| 96 | Zc TRNL 10(3)* | ZcTRL35 | 9 | |
| 97 | Zc TRNL 10(4)* | ZcTRL36 | 9 | |
| 98 | Zc TRNL 11(1) | ZcTRL37 | 9 | |
| 99 | Zc TRNL 11(2) | ZcTRL38 | 9 | |
| 100 | Zc TRNL 11(3) | ZcTRL39 | 9 | |
| 101 | Zc TRNL 11(4) | ZcTRL40 | 9 | |
| 102 | Zc TRNL 12(1) | ZcTRL41 | 9 | |
| 103 | Zc TRNL 12(2) | ZcTRL42 | 9 | |
| 104 | Zc TRNL 12(3) | ZcTRL43 | 9 | |
| 105 | Zc TRNL 12(4) | ZcTRL44 | 9 | |
| 106 | Zc TRNL 13(1) | ZcTRL45 | 5 | |
| 107 | Zc TRNL 13(2)* | ZcTRL46 | 9 | |
| 108 | Zc TRNL 13(3) | ZcTRL47 | 9 | |
| 109 | Zc TRNL 13(4) | ZcTRL48 | 9 | |
| 110 | Zc TRNL 14(1) | ZcTRL49 | 9 | |
| 111 | Zc TRNL 14(2) | ZcTRL50 | 9 | |
| 112 | Zc TRNL 14(3) | ZcTRL51 | 9 | |
| 113 | Zc TRNL 14(4) | ZcTRL52 | 9 | |
| 114 | Zc TRNL 16(1) | ZcTRL53 | 9 | |
| 115 | Zc TRNL 16(2) | ZcTRL54 | 9 | |
| 116 | Zc TRNL 16(3) | ZcTRL55 | 9 | |
| 117 | Zc TRNL 16(4) | ZcTRL56 | 9 | |
| 118 | Zc TRNL 19(1)* | ZcTRL57 | 9 | |
| 119 | Zc TRNL 19(2) | ZcTRL58 | 9 | |
| 120 | Zc TRNL 19(3) | ZcTRL59 | 9 | |
| 121 | Zc TRNL 19(4) | ZcTRL60 | 9 | |
|  |  |  |  | |
| III Population: ZcCU (Collecting site Calicut) | | | | |
| 122 | Zc CU 3(1) | ZcCU01 | 9 | |
| 123 | Zc CU 3(2) | ZcCU02 | 9 | |
| 124 | Zc CU 3(3) | ZcCU03 | 9 | |
| 125 | Zc CU 3(4)* | ZcCU04 | 9 | |
| 126 | Zc CU 5(1) | ZcCU05 | 9 | |
| 127 | Zc CU 5(2) | ZcCU06 | 9 | |
| 128 | Zc CU 5(3) | ZcCU07 | 9 | |
| 129 | Zc CU 5(4)* | ZcCU08 | 9 | |
| 130 | Zc CU 6(1) | ZcCU09 | 9 | |
| 131 | Zc CU 6(2) | ZcCU10 | 9 | |
| 132 | Zc CU 6(3) | ZcCU11 | 9 | |
| 133 | Zc CU 6(4) | ZcCU12 | 9 | |
| 134 | Zc CU 8(1) | ZcCU13 | 7 | |
| 135 | Zc CU 8(2) | ZcCU14 | 9 | |
| 136 | Zc CU 8(3) | ZcCU15 | 9 | |
| 137 | Zc CU 8(4) | ZcCU16 | 5 | |
| 138 | Zc CU 9(1) | ZcCU17 | 9 | |
| 139 | Zc CU 9(2) | ZcCU18 | 9 | |
| 140 | Zc CU 9(3) | ZcCU19 | 9 | |
| 141 | Zc CU 9(4) | ZcCU20 | 9 | |
| 142 | Zc CU 10(1) | ZcCU21 | 9 | |
| 143 | Zc CU 10(2) | ZcCU22 | 9 | |
| 144 | Zc CU 10(3)* | ZcCU23 | 9 | |
| 145 | Zc CU 10(4) | ZcCU24 | 9 | |
| 146 | Zc CU 11(1) | ZcCU25 | 9 | |
| 147 | Zc CU 11(2) | ZcCU26 | 9 | |
| 148 | Zc CU 11(3) | ZcCU27 | 9 | |
| 149 | Zc CU 11(4)* | ZcCU28 | 9 | |
| 150 | Zc CU 12(1) | ZcCU29 | 9 | |
| 151 | Zc CU 12(2) | ZcCU30 | 9 | |
| 152 | Zc CU 12(3) | ZcCU31 | 9 | |
| 153 | Zc CU 12(4)* | ZcCU32 | 9 | |
| 154 | Zc CU 13(1)* | ZcCU33 | 9 | |
| 155 | Zc CU 13(2) | ZcCU34 | 9 | |
| 156 | Zc CU 13(3) | ZcCU35 | 9 | |
| 157 | Zc CU 13(4)* | ZcCU36 | 9 | |
| 158 | Zc CU 18(1) | ZcCU37 | 9 | |
| 159 | Zc CU 18(2) | ZcCU38 | 9 | |
| 160 | Zc CU 18(3) | ZcCU39 | 9 | |
| 161 | Zc CU 18(4) | ZcCU40 | 9 | |
| 162 | Zc CU 19(1)* | ZcCU41 | 9 | |
| 163 | Zc CU 19(2) | ZcCU42 | 9 | |
| 164 | Zc CU 19(3) | ZcCU43 | 9 | |
| 165 | Zc CU 19(4) | ZcCU44 | 9 | |
| 166 | Zc CU 20(1) | ZcCU45 | 9 | |
| 167 | Zc CU 20(2) | ZcCU46 | 9 | |
| 168 | Zc CU 20(3)* | ZcCU47 | 9 | |
| 169 | Zc CU 20(4) | ZcCU48 | 9 | |
| 170 | Zc CU 21(1)* | ZcCU49 | 7 | |
| 171 | Zc CU 21(2) | ZcCU50 | 9 | |
| 172 | Zc CU 21(3) | ZcCU51 | 3 | |
| 173 | Zc CU 21(4) | ZcCU52 | 9 | |
| 174 | Zc CU 22(1) | ZcCU53 | 9 | |
| 175 | Zc CU 22(2) | ZcCU54 | 9 | |
| 176 | Zc CU 22(3) | ZcCU55 | 9 | |
| 177 | Zc CU 22(4) | ZcCU56 | 9 | |
| 178 | Zc CU 24(1) | ZcCU57 | 9 | |
| 179 | Zc CU 24(2) | ZcCU58 | 7 | |
| 180 | Zc CU 24(3) | ZcCU59 | 9 | |
| 181 | Zc CU 24(4) | ZcCU60 | 9 | |
|  |  |  |  | |
| IV Population: ZcVRR (Collecting site Varavoor) | | | | |
| 182 | Zc VRVR 14 (1) | ZcVRR01 | 9 | |
| 183 | Zc VRVR 14 (2) | ZcVRR02 | 9 | |
| 184 | Zc VRVR 14 (3) | ZcVRR03 | 9 | |
| 185 | Zc VRVR 14 (4) | ZcVRR04 | 9 | |
| 186 | Zc VRVR 6 (1) | ZcVRR05 | 9 | |
| 187 | Zc VRVR 6 (2) | ZcVRR06 | 9 | |
| 188 | Zc VRVR 6 (3) | ZcVRR07 | 9 | |
| 189 | Zc VRVR 6 (4) | ZcVRR08 | 9 | |
| 190 | Zc VRVR 8 (1) | ZcVRR09 | 9 | |
| 191 | Zc VRVR 8 (2)* | ZcVRR10 | 9 | |
| 192 | Zc VRVR 8 (3) | ZcVRR11 | 9 | |
| 193 | Zc VRVR 8 (4) | ZcVRR12 | 9 | |
| 194 | Zc VRVR 9 (1) | ZcVRR13 | 9 | |
| 195 | Zc VRVR 9 (2) | ZcVRR14 | 9 | |
| 196 | Zc VRVR 9 (3) | ZcVRR15 | 9 | |
| 197 | Zc VRVR 9 (4) | ZcVRR16 | 9 | |
| 198 | Zc VRVR 10 (1) | ZcVRR17 | 9 | |
| 199 | Zc VRVR 10 (2)* | ZcVRR18 | 9 | |
| 200 | Zc VRVR 10 (3) | ZcVRR19 | 9 | |
| 201 | Zc VRVR 10 (4) | ZcVRR20 | 9 | |
| 202 | Zc VRVR 12 (1) | ZcVRR21 | 9 | |
| 203 | Zc VRVR 12 (2) | ZcVRR22 | 9 | |
| 204 | Zc VRVR 12 (3)* | ZcVRR23 | 9 | |
| 205 | Zc VRVR 12 (4) | ZcVRR24 | 9 | |
| 206 | Zc VRVR 15 (1) | ZcVRR25 | 9 | |
| 207 | Zc VRVR 15 (2)* | ZcVRR26 | 9 | |
| 208 | Zc VRVR 15 (3)* | ZcVRR27 | 9 | |
| 209 | Zc VRVR 15 (4) | ZcVRR28 | 9 | |
| 210 | Zc VRVR 16 (1)* | ZcVRR29 | 9 | |
| 211 | Zc VRVR 16 (2) | ZcVRR30 | 9 | |
| 212 | Zc VRVR 16 (3) | ZcVRR31 | 9 | |
| 213 | Zc VRVR 16 (4) | ZcVRR32 | 9 | |
| 214 | Zc VRVR 17 (1)* | ZcVRR33 | 9 | |
| 215 | Zc VRVR 17 (2) | ZcVRR34 | 9 | |
| 216 | Zc VRVR 17 (3) | ZcVRR35 | 9 | |
| 217 | Zc VRVR 17 (4) | ZcVRR36 | 9 | |
| 218 | Zc VRVR 20 (1)* | ZcVRR37 | 9 | |
| 219 | Zc VRVR 20 (2) | ZcVRR38 | 9 | |
| 220 | Zc VRVR 20 (3) | ZcVRR39 | 9 | |
| 221 | Zc VRVR 20 (4) | ZcVRR40 | 9 | |
| 222 | Zc VRVR 21 (1) | ZcVRR41 | 9 | |
| 223 | Zc VRVR 21 (2) | ZcVRR42 | 9 | |
| 224 | Zc VRVR 21 (3) | ZcVRR43 | 9 | |
| 225 | Zc VRVR 21 (4) | ZcVRR44 | 9 | |
| 226 | Zc VRVR 22 (1) | ZcVRR45 | 9 | |
| 227 | Zc VRVR 22 (2) | ZcVRR46 | 9 | |
| 228 | Zc VRVR 22 (3) | ZcVRR47 | 9 | |
| 229 | Zc VRVR 22 (4) | ZcVRR48 | 9 | |
| 230 | Zc VRVR 23 (1) | ZcVRR49 | 9 | |
| 231 | Zc VRVR 23 (2) | ZcVRR50 | 9 | |
| 232 | Zc VRVR 23 (3) | ZcVRR51 | 9 | |
| 233 | Zc VRVR 23 (4) | ZcVRR52 | 9 | |
| 234 | Zc VRVR 24 (1) | ZcVRR53 | 9 | |
| 235 | Zc VRVR 24 (2) | ZcVRR54 | 9 | |
| 236 | Zc VRVR 24 (3)* | ZcVRR55 | 9 | |
| 237 | Zc VRVR 24 (4) | ZcVRR56 | 9 | |
| 238 | Zc VRVR 25 (1) | ZcVRR57 | 9 | |
| 239 | Zc VRVR 25 (2) | ZcVRR58 | 9 | |
| 240 | Zc VRVR 25 (3)* | ZcVRR59 | 9 | |
| 241 | Zc VRVR 25 (4) | ZcVRR60 | 9 | |
|  |  |  |  | |
| V Population: ZcPVD (Collecting site Poovathodu) | | | | |
| 242 | Zc PVTD 4(1) | ZcPVD01 | 5 | |
| 243 | Zc PVTD 4(2) | ZcPVD02 | 9 | |
| 244 | Zc PVTD 4(3) | ZcPVD03 | 7 | |
| 245 | Zc PVTD 4(4) | ZcPVD04 | 9 | |
| 246 | Zc PVTD 5(1) | ZcPVD05 | 9 | |
| 247 | Zc PVTD 5(2)* | ZcPVD06 | 9 | |
| 248 | Zc PVTD 5(3) | ZcPVD07 | 9 | |
| 249 | Zc PVTD 5(4) | ZcPVD08 | 9 | |
| 250 | Zc PVTD 6(1) | ZcPVD09 | 7 | |
| 251 | Zc PVTD 6(2) | ZcPVD10 | 5 | |
| 252 | Zc PVTD 6(3) | ZcPVD11 | 3 | |
| 253 | Zc PVTD 6(4) | ZcPVD12 | 9 | |
| 254 | Zc PVTD 7(1) | ZcPVD13 | 9 | |
| 255 | Zc PVTD 7(2)* ¥ | ZcPVD14 | 9 | |
| 256 | Zc PVTD 7(3) | ZcPVD15 | 9 | |
| 257 | Zc PVTD 7(4) | ZcPVD16 | 9 | |
| 258 | Zc PVTD 8(1) | ZcPVD17 | 9 | |
| 259 | Zc PVTD 8(2) | ZcPVD18 | 7 | |
| 260 | Zc PVTD 8(3) | ZcPVD19 | 9 | |
| 261 | Zc PVTD 8(4) | ZcPVD20 | 3 | |
| 262 | Zc PVTD 9(1) | ZcPVD21 | 9 | |
| 263 | Zc PVTD 9(2) | ZcPVD22 | 7 | |
| 264 | Zc PVTD 9(3)* | ZcPVD23 | 9 | |
| 265 | Zc PVTD 9(4)* | ZcPVD24 | 9 | |
| 266 | Zc PVTD 11(1) | ZcPVD25 | 9 | |
| 267 | Zc PVTD 11(2) | ZcPVD26 | 5 | |
| 268 | Zc PVTD 11(3) | ZcPVD27 | 9 | |
| 269 | Zc PVTD 11(4) | ZcPVD28 | 9 | |
| 270 | Zc PVTD 10(1)* | ZcPVD29 | 9 | |
| 271 | Zc PVTD 10(2) | ZcPVD30 | 9 | |
| 272 | Zc PVTD 10(3) | ZcPVD31 | 9 | |
| 273 | Zc PVTD 10(4) | ZcPVD32 | 9 | |
| 274 | Zc PVTD 13(1) | ZcPVD33 | 9 | |
| 275 | Zc PVTD 13(2) | ZcPVD34 | 9 | |
| 276 | Zc PVTD 13(3) | ZcPVD35 | 9 | |
| 277 | Zc PVTD 13(4) | ZcPVD36 | 9 | |
| 278 | Zc PVTD 16(1) | ZcPVD37 | 9 | |
| 279 | Zc PVTD 16(2) | ZcPVD38 | 7 | |
| 280 | Zc PVTD 16(3)* | ZcPVD39 | 9 | |
| 281 | Zc PVTD 16(4) | ZcPVD40 | 9 | |
| 282 | Zc PVTD 17(1) | ZcPVD41 | 9 | |
| 283 | Zc PVTD 17(2) | ZcPVD42 | 7 | |
| 284 | Zc PVTD 17(3) | ZcPVD43 | 9 | |
| 285 | Zc PVTD 17(4) | ZcPVD44 | 9 | |
| 286 | Zc PVTD 19(1) | ZcPVD45 | 9 | |
| 287 | Zc PVTD 19(2) | ZcPVD46 | 0 | |
| 288 | Zc PVTD 19(3) | ZcPVD47 | 9 | |
| 289 | Zc PVTD 19(4) | ZcPVD48 | 9 | |
| 290 | Zc PVTD 22(1) | ZcPVD49 | 9 | |
| 291 | Zc PVTD 22(2) | ZcPVD50 | 9 | |
| 292 | Zc PVTD 22(3) | ZcPVD51 | 9 | |
| 293 | Zc PVTD 22(4) | ZcPVD52 | 9 | |
| 294 | Zc PVTD 24(1) | ZcPVD53 | 9 | |
| 295 | Zc PVTD 24(2) | ZcPVD54 | 3 | |
| 296 | Zc PVTD 24(3)* | ZcPVD55 | 9 | |
| 297 | Zc PVTD 24(4) | ZcPVD56 | 7 | |
| 298 | Zc PVTD 18(1) | ZcPVD57 | 9 | |
| 299 | Zc PVTD 18(2)* | ZcPVD58 | 9 | |
| 300 | Zc PVTD 18(3) | ZcPVD59 | 9 | |
| 301 | Zc PVTD 18(4) | ZcPVD60 | 9 | |
| 302 | Zc PVTD 25(3)^*#^ | ZcPVD61 |  | |
| 303 | Zc PVTD 25(4)^*#^ | ZcPVD62 |  | |
|  |  |  |  | |
| Sl. No. | Accession Number | Code No. | Disease Score | |
| **S2c** *Z*. *zerumbet* | | | | |
|  |  |  |  | |
| I Population: ZzKNR (Collecting site Kannur) | | | | |
| 1 | KNR135* | ZzKNR01 | 0 | |
| 2 | KNR137* | ZzKNR02 | 0 | |
| 3 | KNR139*¥ | ZzKNR03 | 0 | |
| 4 | KNR141* | ZzKNR04 | 0 | |
| 5 | KNR142*¥ | ZzKNR05 | 0 | |
| 6 | KNR144* | ZzKNR06 | 0 | |
| 7 | KNR147* | ZzKNR07 | 0 | |
| 8 | KNR149* | ZzKNR08 | 0 | |
| 9 | KNR151* | ZzKNR09 | 0 | |
| 10 | KNR152* | ZzKNR10 | 0 | |
| 11 | KNR154 | ZzKNR11 | 0 | |
| 12 | KNR158 | ZzKNR12 | 0 | |
| 13 | KNR160 | ZzKNR13 | 0 | |
| 14 | KNR162 | ZzKNR14 | 0 | |
| 15 | KNR163 | ZzKNR15 | 0 | |
| 16 | KNR166 | ZzKNR16 | 0 | |
| 17 | KNR167 | ZzKNR17 | 0 | |
| 18 | KNR356 | ZzKNR18 | 1 | |
| 19 | KNR358 | ZzKNR19 | 1 | |
| 20 | KNR359 | ZzKNR20 | 0 | |
| 21 | KNR361 | ZzKNR21 | 0 | |
| 22 | KNR364 | ZzKNR22 | 0 | |
| 23 | KNR366 | ZzKNR23 | 0 | |
| 24 | KNR368 | ZzKNR24 | 0 | |
| 25 | KNR371 | ZzKNR25 | 0 | |
| 26 | KNR382 | ZzKNR26 | 0 | |
| 27 | KNR386 | ZzKNR27 | 0 | |
| 28 | KNR389 | ZzKNR28 | 5 | |
| 29 | KNR394 | ZzKNR29 | 1 | |
| 30 | KNR413 | ZzKNR30 | 0 | |
|  |  |  |  | |
| II Population: ZzCLT (Collecting site Calicut) | | | | |
| 31 | CLT169* | ZzCLT01 | 0 | |
| 32 | CLT171* | ZzCLT02 | 0 | |
| 33 | CLT172* | ZzCLT03 | 0 | |
| 34 | CLT174* | ZzCLT04 | 1 | |
| 35 | CLT175*¥ | ZzCLT05 | 0 | |
| 36 | CLT178* | ZzCLT06 | 0 | |
| 37 | CLT180* | ZzCLT07 | 0 | |
| 38 | CLT183* | ZzCLT08 | 0 | |
| 39 | CLT184* | ZzCLT09 | 0 | |
| 40 | CLT186 | ZzCLT10 | 0 | |
| 41 | CLT188 | ZzCLT11 | 0 | |
| 42 | CLT191 | ZzCLT12 | 0 | |
| 43 | CLT193 | ZzCLT13 | 0 | |
| 44 | CLT195 | ZzCLT14 | 0 | |
| 45 | CLT197 | ZzCLT15 | 0 | |
| 46 | CLT421 | ZzCLT16 | 0 | |
| 47 | CLT422 | ZzCLT17 | 3 | |
| 48 | CLT424 | ZzCLT18 | 0 | |
| 49 | CLT425 | ZzCLT19 | 0 | |
| 50 | CLT428 | ZzCLT20 | 0 | |
| 51 | CLT433 | ZzCLT21 | 0 | |
| 52 | CLT442 | ZzCLT22 | 0 | |
| 53 | CLT444 | ZzCLT23 | 0 | |
| 54 | CLT449 | ZzCLT24 | 0 | |
| 55 | CLT450 | ZzCLT25 | 1 | |
| 56 | CLT452 | ZzCLT26 | 0 | |
| 57 | CLT454 | ZzCLT27 | 0 | |
| 58 | CLT460 | ZzCLT28 | 0 | |
| 59 | CLT461 | ZzCLT29 | 0 | |
| 60 | CLT462 | ZzCLT30 | 0 | |
| 61 | CLT478 | ZzCLT31 | 0 | |
| 62 | CLT221-6^*#^¥ | ZzCLT32 |  | |
|  |  |  |  | |
| III Population: ZzPZR (Collecting site Pazhayannur) | | | | |
| 63 | PZR301* | ZzPZR01 | 0 | |
| 64 | PZR302* | ZzPZR02 | 0 | |
| 65 | PZR305*¥ | ZzPZR03 | 0 | |
| 66 | PZR307* | ZzPZR04 | 0 | |
| 67 | PZR308 | ZzPZR05 | 0 | |
| 68 | PZR309-1*¥ | ZzPZR06 | 0 | |
| 69 | PZR310 | ZzPZR07 | 0 | |
| 70 | PZR311* | ZzPZR08 | 0 | |
| 71 | PZR315* | ZzPZR09 | 0 | |
| 72 | PZR316* | ZzPZR10 | 0 | |
| 73 | PZR319* | ZzPZR11 | 0 | |
| 74 | PZR321* | ZzPZR12 | 0 | |
| 75 | PZR323 | ZzPZR13 | 0 | |
| 76 | PZR480 | ZzPZR14 | 1 | |
| 77 | PZR483 | ZzPZR15 | 0 | |
| 78 | PZR485 | ZzPZR16 | 0 | |
| 79 | PZR486 | ZzPZR17 | 0 | |
| 80 | PZR488 | ZzPZR18 | 0 | |
| 81 | PZR492 | ZzPZR19 | 0 | |
| 82 | PZR493 | ZzPZR20 | 0 | |
| 83 | PZR496 | ZzPZR21 | 0 | |
| 84 | PZR497 | ZzPZR22 | 0 | |
| 85 | PZR502 | ZzPZR23 | 3 | |
| 86 | PZR504 | ZzPZR24 | 0 | |
| 87 | PZR505 | ZzPZR25 | 0 | |
| 88 | PZR506 | ZzPZR26 | 0 | |
| 89 | PZR507 | ZzPZR27 | 0 | |
| 90 | PZR520 | ZzPZR28 | 0 | |
| 91 | PZR526 | ZzPZR29 | 0 | |
| 92 | PZR532-1 | ZzPZR30 | 7 | |
| 93 | PZR533-1 | ZzPZR31 | 0 | |
|  |  |  |  | |
| IV Population: ZzKLD (Collecting site Kalady) | | | | |
| 94 | KLD17 | ZzKLD01 | 0 | |
| 95 | KLD20 | ZzKLD02 | 0 | |
| 96 | KLD21 | ZzKLD03 | 0 | |
| 97 | KLD22 | ZzKLD04 | 1 | |
| 98 | KLD25 | ZzKLD05 | 0 | |
| 99 | KLD26 | ZzKLD06 | 1 | |
| 100 | KLD27 | ZzKLD07 | 0 | |
| 101 | KLD31 | ZzKLD08 | 0 | |
| 102 | KLD32 | ZzKLD09 | 0 | |
| 103 | KLD35 | ZzKLD10 | 3 | |
| 104 | KLD36 | ZzKLD11 | 0 | |
| 105 | KLD47 | ZzKLD12 | 0 | |
| 106 | KLD48 | ZzKLD13 | 0 | |
| 107 | KLD49 | ZzKLD14 | 0 | |
| 108 | KLD51 | ZzKLD15 | 3 | |
| 109 | KLD54 | ZzKLD16 | 1 | |
| 110 | KLD58 | ZzKLD17 | 0 | |
| 111 | KLD59 | ZzKLD18 | 3 | |
| 112 | KLD61 | ZzKLD19 | 0 | |
| 113 | KLD69 | ZzKLD20 | 1 | |
| 114 | KLD74 | ZzKLD21 | 0 | |
| 115 | KLD326* | ZzKLD22 | 0 | |
| 116 | KLD327* | ZzKLD23 | 3 | |
| 117 | KLD332* | ZzKLD24 | 0 | |
| 118 | KLD335* | ZzKLD25 | 0 | |
| 119 | KLD339*¥ | ZzKLD26 | 0 | |
| 120 | KLD341* | ZzKLD27 | 0 | |
| 121 | KLD345* | ZzKLD28 | 0 | |
| 122 | KLD346* | ZzKLD29 | 0 | |
| 123 | KLD350* | ZzKLD30 | 1 | |
| 124 | KLD353 | ZzKLD31 | 1 | |
| 125 | KLD226-4^*#^¥ | ZzKLD32 |  | |
|  |  |  |  | |
| V Population: ZzPVD (Collecting site Poovathodu) | | | | |
| 126 | PVD17 | ZzPVD01 | 0 | |
| 127 | PVD21 | ZzPVD02 | 0 | |
| 128 | PVD22 | ZzPVD03 | 0 | |
| 129 | PVD27 | ZzPVD04 | 0 | |
| 130 | PVD29 | ZzPVD05 | 0 | |
| 131 | PVD31 | ZzPVD06 | 0 | |
| 132 | PVD32 | ZzPVD07 | 0 | |
| 133 | PVD34 | ZzPVD08 | 0 | |
| 134 | PVD35 | ZzPVD09 | 1 | |
| 135 | PVD38 | ZzPVD10 | 3 | |
| 136 | PVD41 | ZzPVD11 | 0 | |
| 137 | PVD50 | ZzPVD12 | 0 | |
| 138 | PVD52 | ZzPVD13 | 0 | |
| 139 | PVD54 | ZzPVD14 | 0 | |
| 140 | PVD55 | ZzPVD15 | 0 | |
| 141 | PVD56 | ZzPVD16 | 0 | |
| 142 | PVD57 | ZzPVD17 | 0 | |
| 143 | PVD58 | ZzPVD18 | 0 | |
| 144 | PVD64 | ZzPVD19 | 0 | |
| 145 | PVD66 | ZzPVD20 | 0 | |
| 146 | PVD69 | ZzPVD21 | 0 | |
| 147 | PVD74 | ZzPVD22 | 0 | |
| 148 | PVD75 | ZzPVD23 | 0 | |
| 149 | PVD92 | ZzPVD24 | 0 | |
| 150 | PVD274* | ZzPVD25 | 0 | |
| 151 | PVD278*¥ | ZzPVD26 | 0 | |
| 152 | PVD285*¥ | ZzPVD27 | 0 | |
| 153 | PVD286 | ZzPVD28 | 0 | |
| 154 | PVD293 | ZzPVD29 | 0 | |
| 155 | PVD294 | ZzPVD30 | 0 | |
| 156 | PVD224-1^*#^ | ZzPVD31 |  | |
| 157 | PVD224-2^*#^ | ZzPVD32 |  | |
| 158 | PVD224-3^*#^ | ZzPVD33 |  | |
| 159 | PVD224-5^*#^ | ZzPVD34 |  | |
| 160 | PVD224-7^*#^ | ZzPVD35 |  | |
| 161 | PVD224-8^*#^ | ZzPVD36 |  | |
| 162 | PVD224-10^*#^ | ZzPVD37 |  | |
|  |  |  |  | |
| VI Population: ZzKTK (Collecting site Kottarakara) | | | | |
| 163 | KTK200 | ZzKTK01 | 0 | |
| 164 | KTK202* | ZzKTK02 | 0 | |
| 165 | KTK203 | ZzKTK03 | 0 | |
| 166 | KTK205* | ZzKTK04 | 0 | |
| 167 | KTK215* | ZzKTK05 | 0 | |
| 168 | KTK217* | ZzKTK06 | 0 | |
| 169 | KTK219* | ZzKTK07 | 0 | |
| 170 | KTK220* | ZzKTK08 | 0 | |
| 171 | KTK222* | ZzKTK09 | 0 | |
| 172 | KTK227* | ZzKTK10 | 0 | |
| 173 | KTK228 | ZzKTK11 | 0 | |
| 174 | KTK230 | ZzKTK12 | 0 | |
| 175 | KTK234 | ZzKTK13 | 0 | |
| 176 | KTK623 | ZzKTK14 | 0 | |
| 177 | KTK627 | ZzKTK15 | 0 | |
| 178 | KTK628 | ZzKTK16 | 3 | |
| 179 | KTK634 | ZzKTK17 | 0 | |
| 180 | KTK637 | ZzKTK18 | 7 | |
| 181 | KTK638 | ZzKTK19 | 0 | |
| 182 | KTK644 | ZzKTK20 | 0 | |
| 183 | KTK648 | ZzKTK21 | 0 | |
| 184 | KTK653 | ZzKTK22 | 3 | |
| 185 | KTK654 | ZzKTK23 | 0 | |
| 186 | KTK655 | ZzKTK24 | 0 | |
| 187 | KTK657 | ZzKTK25 | 0 | |
| 188 | KTK658 | ZzKTK26 | 0 | |
| 189 | KTK661 | ZzKTK27 | 0 | |
| 190 | KTK663 | ZzKTK28 | 0 | |
| 191 | KTK667 | ZzKTK29 | 0 | |
| 192 | KTK222-7^*#^¥ | ZzKTK30 |  | |
| 193 | KTK222-8^*#^¥ | ZzKTK31 |  | |
|  |  |  |  | |
| VII Population: ZzPNR (Collecting site Punalur) | | | | |
| 194 | PNR83 | ZzPNR01 | 0 | |
| 195 | PNR87*¥ | ZzPNR02 | 0 | |
| 196 | PNR90 | ZzPNR03 | 0 | |
| 197 | PNR100* | ZzPNR04 | 0 | |
| 198 | PNR103 | ZzPNR05 | 0 | |
| 199 | PNR104*¥ | ZzPNR06 | 1 | |
| 200 | PNR108* | ZzPNR07 | 0 | |
| 201 | PNR116* | ZzPNR08 | 0 | |
| 202 | PNR119 | ZzPNR09 | 0 | |
| 203 | PNR123* | ZzPNR10 | 0 | |
| 204 | PNR127*¥ | ZzPNR11 | 0 | |
| 205 | PNR130* | ZzPNR12 | 0 | |
| 206 | PNR131 | ZzPNR13 | 0 | |
| 207 | PNR133 | ZzPNR14 | 0 | |
| 208 | PNR544 | ZzPNR15 | 0 | |
| 209 | PNR557 | ZzPNR16 | 0 | |
| 210 | PNR561 | ZzPNR17 | 0 | |
| 211 | PNR569 | ZzPNR18 | 0 | |
| 212 | PNR570 | ZzPNR19 | 0 | |
| 213 | PNR572 | ZzPNR20 | 0 | |
| 214 | PNR576 | ZzPNR21 | 0 | |
| 215 | PNR578 | ZzPNR22 | 3 | |
| 216 | PNR581 | ZzPNR23 | 7 | |
| 217 | PNR582 | ZzPNR24 | 0 | |
| 218 | PNR583 | ZzPNR25 | 0 | |
| 219 | PNR586 | ZzPNR26 | 0 | |
| 220 | PNR587 | ZzPNR27 | 0 | |
| 221 | PNR588 | ZzPNR28 | 0 | |
| 222 | PNR593 | ZzPNR29 | 0 | |
| 223 | PNR595 | ZzPNR30 | 0 | |
| 224 | PNR602 | ZzPNR31 | 0 | |
| 225 | PNR219-6^*#^ | ZzPNR32 |  | |
| 226 | PNR219-13^*#^ | ZzPNR33 |  | |
|  |  |  |  | |
| VIII Population: ZzKLZ (Collecting site Kulathupuzha) | | | | |
| 227 | Zz KLPZ-1(1) | ZzKLZ01 | 9 | |
| 228 | Zz KLPZ-1(3) | ZzKLZ02 | 9 | |
| 229 | Zz KLPZ-1(4)* | ZzKLZ03 | 9 | |
| 230 | Zz KLPZ-2(1) | ZzKLZ04 | 7 | |
| 231 | Zz KLPZ-2(2)* | ZzKLZ05 | 9 | |
| 232 | Zz KLPZ-2(3)* | ZzKLZ06 | 3 | |
| 233 | Zz KLPZ-2(4) | ZzKLZ07 | 5 | |
| 234 | Zz KLPZ-3(1) | ZzKLZ08 | 7 | |
| 235 | Zz KLPZ-3(2)* | ZzKLZ09 | 0 | |
| 236 | Zz KLPZ-3(3) | ZzKLZ10 | 9 | |
| 237 | Zz KLPZ-4(1) | ZzKLZ11 | 5 | |
| 238 | Zz KLPZ-4(2) | ZzKLZ12 | 5 | |
| 239 | Zz KLPZ-4(4)* | ZzKLZ13 | 0 | |
| 240 | Zz KLPZ-5(1) | ZzKLZ14 | 9 | |
| 241 | Zz KLPZ-5(2) | ZzKLZ15 | 9 | |
| 242 | Zz KLPZ-6(1)* | ZzKLZ16 | 0 | |
| 243 | Zz KLPZ-6(2)* | ZzKLZ17 | 3 | |
| 244 | Zz KLPZ-6(3)* | ZzKLZ18 | 0 | |
| 245 | Zz KLPZ-7(1)* | ZzKLZ19 | 3 | |
| 246 | Zz KLPZ-7(2) | ZzKLZ20 | 7 | |
| 247 | Zz KLPZ-7(3)* | ZzKLZ21 | 0 | |
| 248 | Zz KLPZ-8(1) | ZzKLZ22 | 7 | |
| 249 | Zz KLPZ-8(2) | ZzKLZ23 | 7 | |
| 250 | Zz KLPZ-9(1)* | ZzKLZ24 | 9 | |
| 251 | Zz KLPZ-9(2)* | ZzKLZ25 | 9 | |
| 252 | Zz KLPZ-10(1)* | ZzKLZ26 | 9 | |
| 253 | Zz KLPZ-10(2) | ZzKLZ27 | 9 | |
| 254 | Zz KLPZ-10(3) | ZzKLZ28 | 9 | |
| 255 | Zz KLPZ-11(1)* ¥ | ZzKLZ29 | 0 | |
| 256 | Zz KLPZ-11(2) | ZzKLZ30 | 5 | |
| 257 | Zz KLPZ-11(3) | ZzKLZ31 | 9 | |
| 258 | Zz KLPZ-11(4) | ZzKLZ32 | 5 | |
| 259 | Zz KLPZ-12(1) | ZzKLZ33 | 7 | |
| 260 | Zz KLPZ-12(2) | ZzKLZ34 | 7 | |
| 261 | Zz KLPZ-12(3)* | ZzKLZ35 | 0 | |
| 262 | Zz KLPZ-12(4) | ZzKLZ36 | 7 | |
| 263 | Zz KLPZ-13(1) | ZzKLZ37 | 9 | |
| 264 | Zz KLPZ-13(2)* | ZzKLZ38 | 9 | |
| 265 | Zz KLPZ-13(3)* | ZzKLZ39 | 9 | |
| 266 | Zz KLPZ-14(1) | ZzKLZ40 | 9 | |
| 267 | Zz KLPZ-14(2)* | ZzKLZ41 | 9 | |
| 268 | Zz KLPZ-14(3) | ZzKLZ42 | 9 | |
| 269 | Zz KLPZ-15(1) | ZzKLZ43 | 9 | |
| 270 | Zz KLPZ-15(2)* ¥ | ZzKLZ44 | 9 | |
| 271 | Zz KLPZ-15(3)* | ZzKLZ45 | 9 | |
|  |  |  |  | |
| IX Population ; ZzAPR (Collecting site Anappara) | | |  | |
| 272 | Zz TVYD-1(1) | ZzAPR01 | 5 | |
| 273 | Zz TVYD-1(2) | ZzAPR02 | 9 | |
| 274 | Zz TVYD-1(3) | ZzAPR03 | 9 | |
| 275 | Zz TVYD-2(1)* | ZzAPR04 | 9 | |
| 276 | Zz TVYD-2(2)* | ZzAPR05 | 9 | |
| 277 | Zz TVYD-3(1)* | ZzAPR06 | 9 | |
| 278 | Zz TVYD-3(2)* | ZzAPR07 | 0 | |
| 279 | Zz TVYD-4(1)* | ZzAPR08 | 3 | |
| 280 | Zz TVYD-4(2)* | ZzAPR09 | 3 | |
| 281 | Zz TVYD-5(1)* | ZzAPR10 | 9 | |
| 282 | Zz TVYD-5(2)* ¥ | ZzAPR11 | 0 | |
| 283 | Zz TVYD-5(3) | ZzAPR12 | 9 | |
| 284 | Zz TVYD-6(1)* | ZzAPR13 | 0 | |
| 285 | Zz TVYD-6(2) | ZzAPR14 | 5 | |
| 286 | Zz TVYD-6(3) | ZzAPR15 | 0 | |
| 287 | Zz TVYD-6(4) | ZzAPR16 | 9 | |
| 288 | Zz APRA-1(1) | ZzAPR17 | 5 | |
| 289 | Zz APRA-1(2) | ZzAPR18 | 5 | |
| 290 | Zz APRA-1(3) | ZzAPR19 | 7 | |
| 291 | Zz APRA-2(1) | ZzAPR20 | 9 | |
| 292 | Zz APRA-2(2) | ZzAPR21 | 9 | |
| 293 | Zz APRA-2(3) | ZzAPR22 | 7 | |
| 294 | Zz APRA-3(1)* | ZzAPR23 | 5 | |
| 295 | Zz APRA-3(2) | ZzAPR24 | 9 | |
| 296 | Zz APRA-3(3) | ZzAPR25 | 5 | |
| 297 | Zz APRA-3(4) | ZzAPR26 | 9 | |
| 298 | Zz APRA-4(1) | ZzAPR27 | 9 | |
| 299 | Zz APRA-4(2) | ZzAPR28 | 9 | |
| 300 | Zz APRA-4(3)* | ZzAPR29 | 9 | |
| 301 | Zz APRA-4(4) | ZzAPR30 | 7 | |
| 302 | Zz APRA-5(1) | ZzAPR31 | 9 | |
| 303 | Zz APRA-5(2) | ZzAPR32 | 3 | |
| 304 | Zz APRA-5(3) | ZzAPR33 | 3 | |
| 305 | Zz APRA-5(4) | ZzAPR34 | 7 | |
| 306 | Zz APRA-6(1)* | ZzAPR35 | 9 | |
| 307 | Zz APRA-6(2) | ZzAPR36 | 9 | |
| 308 | Zz APRA-6(3)* | ZzAPR37 | 3 | |
| 309 | Zz APRA-7(1) | ZzAPR38 | 5 | |
| 310 | Zz APRA-7(2) | ZzAPR39 | 7 | |
| 311 | Zz APRA-7(3) | ZzAPR40 | 7 | |
| 312 | Zz APRA-7(4) | ZzAPR41 | 7 | |
| 313 | Zz APRA-8(1) | ZzAPR42 | 7 | |
| 314 | Zz APRA-8(2) | ZzAPR43 | 7 | |
| 315 | Zz APRA-8(3)* | ZzAPR44 | 9 | |
| 316 | Zz APRA-8(4) | ZzAPR45 | 9 | |
| 317 | Zz APRA-9(1)* | ZzAPR46 | 9 | |
| 318 | Zz APRA-9(2) | ZzAPR47 | 9 | |
| 319 | Zz APRA-9(3)* | ZzAPR48 | 9 | |
| 320 | Zz APRA-10(1) | ZzAPR49 | 9 | |
| 321 | Zz APRA-10(2) | ZzAPR50 | 9 | |
| 322 | Zz APRA-10(3)* | ZzAPR51 | 9 | |
| 323 | Zz APRA-11(1) | ZzAPR52 | 7 | |
| 324 | Zz APRA-11(2)* | ZzAPR53 | 3 | |
| 325 | Zz APRA-11(3) | ZzAPR54 | 5 | |
| 326 | Zz APRA-11(4) | ZzAPR55 | 7 | |
| 327 | Zz APRA-12(1)* | ZzAPR56 | 3 | |
| 328 | Zz APRA-12(2)* ¥ | ZzAPR57 | 3 | |
|  |  |  |  | |
| X Population: ZzKLR (Collecting site Kallar) | | | | |
| 329 | (10)Zz PNMD-1(1) | ZzKLR01 | | 9 |
| 330 | (10)Zz PNMD-1(2)* | ZzKLR02 | | 3 |
| 331 | (10)Zz PNMD-1(3) | ZzKLR03 | | 9 |
| 332 | (9)Zz PNMD-2(1) | ZzKLR04 | | 7 |
| 333 | (9)Zz PNMD-2(2) | ZzKLR05 | | 7 |
| 334 | (9)Zz PNMD-2(3) | ZzKLR06 | | 7 |
| 335 | (9)Zz PNMD-2(4) | ZzKLR07 | | 7 |
| 336 | (9)Zz PNMD-3(1) | ZzKLR08 | | 9 |
| 337 | (9)Zz PNMD-3(2) | ZzKLR09 | | 9 |
| 338 | (9)Zz PNMD-3(3) | ZzKLR10 | | 9 |
| 339 | (9)Zz PNMD-4(1) | ZzKLR11 | | 9 |
| 340 | (9)Zz PNMD-4(2)* | ZzKLR12 | | 9 |
| 341 | (9)Zz PNMD-4(3) | ZzKLR13 | | 9 |
| 342 | (9)Zz PNMD-5(1) | ZzKLR14 | | 7 |
| 343 | (9)Zz PNMD-5(2) | ZzKLR15 | | 9 |
| 344 | (9)Zz PNMD-5(3) | ZzKLR16 | | 3 |
| 345 | (9)Zz PNMD-5(4) | ZzKLR17 | | 7 |
| 346 | (9)Zz PNMD-6(1) | ZzKLR18 | | 9 |
| 347 | (9)Zz PNMD-6(2) | ZzKLR19 | | 9 |
| 348 | (9)Zz PNMD-6(3) | ZzKLR20 | | 9 |
| 349 | (9)Zz PNMD-6(4) | ZzKLR21 | | 9 |
| 350 | (9)Zz PNMD-7(1) | ZzKLR22 | | 9 |
| 351 | (9)Zz PNMD-7(2) | ZzKLR23 | | 9 |
| 352 | (9)Zz PNMD-7(3) | ZzKLR24 | | 9 |
| 353 | (9)Zz PNMD-8(1) | ZzKLR25 | | 9 |
| 354 | (9)Zz PNMD-8(2) | ZzKLR26 | | 7 |
| 355 | (9)Zz PNMD-8(3)* ¥ | ZzKLR27 | | 5 |
| 356 | (10)Zz PNMD-9(1)* | ZzKLR28 | | 9 |
| 357 | (10)Zz PNMD-9(2)* | ZzKLR29 | | 5 |
| 358 | (10)Zz PNMD-10(1) | ZzKLR30 | | 9 |
| 359 | (10)Zz PNMD-11(1) | ZzKLR31 | | 9 |
| 360 | (10)Zz PNMD-12(1)* | ZzKLR32 | | 9 |
| 361 | (10)Zz PNMD-12(2)* | ZzKLR33 | | 9 |
| 362 | (10)Zz PNMD-13(1) | ZzKLR34 | | 9 |
| 363 | (10)Zz PNMD-13(2)* | ZzKLR35 | | 9 |
| 364 | (10)Zz PNMD-13(3)* | ZzKLR36 | | 9 |
| 365 | (10)Zz PNMD-13(4) | ZzKLR37 | | 9 |
| 366 | (10)Zz PNMD-14(1)* | ZzKLR38 | | 9 |
| 367 | (10)Zz PNMD-14(2)* | ZzKLR39 | | 9 |
| 368 | (10)Zz PNMD-14(3)* | ZzKLR40 | | 9 |
| 369 | (10)Zz PNMD-15(1) | ZzKLR41 | | 9 |
| 370 | (10)Zz PNMD-15(2) | ZzKLR42 | | 7 |
| 371 | (10)Zz PNMD-15(3)* ¥ | ZzKLR43 | | 9 |
| 372 | (9)Zz PNMD-1(1) | ZzKLR44 | | 9 |
| 373 | (9)Zz PNMD-1(2) | ZzKLR45 | | 9 |
| 374 | (9)Zz PNMD-1(3) | ZzKLR46 | | 9 |
| 375 | (10)Zz PNMD-3(1)* | ZzKLR47 | | 5 |
| 376 | (10)Zz PNMD-3(2) | ZzKLR48 | | 9 |
| 377 | (10)Zz PNMD-2(1)* | ZzKLR49 | | 5 |
| 378 | (10)Zz PNMD-2(2) | ZzKLR50 | | 7 |
| 379 | (10)Zz PNMD-6(1)* | ZzKLR51 | | 0 |
| 380 | (10)Zz PNMD-6(2) | ZzKLR52 | | 9 |
| 381 | (10)Zz PNMD-6(3)* ¥ | ZzKLR53 | | 0 |
| 382 | (10)Zz PNMD-4(1)* | ZzKLR54 | | 3 |
| 383 | (10)Zz PNMD-5(1) | ZzKLR55 | | 7 |
| 384 | (10)Zz PNMD-5(2) | ZzKLR56 | | 7 |
| 385 | (10)Zz PNMD-7(1)* | ZzKLR57 | | 5 |
| 386 | (10)Zz PNMD-8(1)* | ZzKLR58 | | 0 |
| 387 | (10)Zz PNMD-8(2) | ZzKLR59 | | 3 |
| 388 | (10)Zz PNMD-8(3) | ZzKLR60 | | 7 |
| 389 | (10)Zz PNMD-8(4) | ZzKLR61 | | 7 |
| \| * Samples included in AFLP analysis \| \| --- \| \| ^*#^Samples included in AFLP analysis but not in infection study \| | | | | |

¥ Individuals used in initial primer screening and for the estimation for AFLP error rate

**Table S3a** Geographic distance (km) between the collecting sites of *Z. neesanum* populations

|  | ZnTRL | ZnWGN | ZnPMD |
| --- | --- | --- | --- |
| ZnTRL | 0 |  |  |
| ZnWGN | 370 | 0 |  |
| ZnPMD | 521 | 206 | 0 |

**Table S3b** Geographic distance (km) between the collecting sites of *Z. nimmonii* populations

|  | ZcKNR | ZcTRL | ZcCU | ZcVRR | ZzPVD |
| --- | --- | --- | --- | --- | --- |
| ZcKNR | 0 |  |  |  |  |
| ZcTRL | 121 | 0 |  |  |  |
| ZcCU | 114 | 136 | 0 |  |  |
| ZcVRR | 193 | 201 | 82 | 0 |  |
| ZzPVD | 338 | 353 | 225 | 156 | 0 |

**Table S3c** Geographic distance (km) between the collecting sites of *Z. zerumbet* populations

|  | ZzKNR | ZzCLT | ZzPZR | ZzKLD | ZzPVD | ZzKTK | ZzPNR | ZzKLZ | ZzAPR | ZzKLR |
| --- | --- | --- | --- | --- | --- | --- | --- | --- | --- | --- |
| ZzKNR | 0 |  |  |  |  |  |  |  |  |  |
| ZzCLT | 114 | 0 |  |  |  |  |  |  |  |  |
| ZzPZR | 206 | 92 | 0 |  |  |  |  |  |  |  |
| ZzKLD | 265 | 151 | 59 | 0 |  |  |  |  |  |  |
| ZzPVD | 338 | 216 | 124 | 65 | 0 |  |  |  |  |  |
| ZzKTK | 410 | 296 | 204 | 145 | 80 | 0 |  |  |  |  |
| ZzPNR | 420 | 306 | 214 | 155 | 90 | 10 | 0 |  |  |  |
| ZzKLZ | 447 | 333 | 241 | 182 | 117 | 37 | 27 | 0 |  |  |
| ZzAPR | 480 | 366 | 274 | 215 | 150 | 70 | 60 | 33 | 0 |  |
| ZzKLR | 485 | 371 | 279 | 220 | 155 | 75 | 65 | 38 | 5 | 0 |

**Table S4** Primer combinations used for AFLP analysis of *Zingiber* spp. together with the percentage polymorphism obtained for each primer combination

| Species | Primer combinations | Total bands | Number of polymorphic bands |
| --- | --- | --- | --- |
| *Z. officinale* | E-ACA x M-CTT | 50 | 0 |
|  | E-ACC x M-CAA | 38 | 0 |
|  | E-ACT x M-CTA | 54 | 3 |
|  | E-ACT x M-CTC | 36 | 0 |
|  | E-AGC x M-CTC | 25 | 2 |
|  | Total | 203 | 5 |
| *Z. neesanum* | E-ACC x M-CAA | 58 | 37 |
|  | E-ACA x M-CTC | 60 | 41 |
|  | E-ACT x M-CTC | 47 | 37 |
|  | E-AGC x M-CTC | 41 | 25 |
|  | E-ACA x M-CTT | 54 | 21 |
|  | E-ACT x M-CTT | 55 | 31 |
|  | Total | 315 | 192 |
| *Z. nimmonii* | E-ACC x M-CAA | 49 | 33 |
|  | E-ACA x M-CTC | 41 | 13 |
|  | E-ACT x M-CTC | 59 | 31 |
|  | E-AGC x M-CTC | 48 | 27 |
|  | E-ACA x M-CTT | 61 | 28 |
|  | E-ACT x M-CTT | 63 | 25 |
|  | Total | 321 | 157 |
| *Z. zerumbet* | A-ACT x M-CTA | 69 | 69 |
|  | E-AAC x M-CAC | 64 | 56 |
|  | E-ACA x M-CAG | 37 | 37 |
|  | E-ACC x M-CAA | 51 | 48 |
|  | E-ACC x M-CTA | 55 | 48 |
|  | E-ACT x M-CAT | 55 | 54 |
|  | Total | 331 | 312 |

**Table S5** Pair-wise *F*st between the populations and mean *F*st in three *Zingiber* spp. used in the study

**Table S5a** *Z. neesanum*

|  | ZnTRL | ZnWGN | ZnPMD |
| --- | --- | --- | --- |
| ZnTRL | 0 |  |  |
| ZnWGN | 0.83648 | 0 |  |
| ZnPMD | 0.82755 | 0.45033 | 0 |

Mean *F*st = 0.78209 (significant *p <* 0.05)

**Table S5b** *Z. nimmonii*

|  | ZcKNR | ZcTRL | ZcCU | ZcVRR | ZzPVD |
| --- | --- | --- | --- | --- | --- |
| ZcKNR | 0 |  |  |  |  |
| ZcTRL | 0.27304 | 0 |  |  |  |
| ZcCU | 0.42444 | 0.29526 | 0 |  |  |
| ZcVRR | 0.46332 | 0.16645 | 0.31595 | 0 |  |
| ZzPVD | 0.58969 | 0.35328 | 0.45697 | 0.35091 | 0 |

Mean *F*st = 0.37367 (significant *p <* 0.05)

**Table S5c** *Z. zerumbet*

|  | ZzKNR | ZzCLT | ZzPZR | ZzKLD | ZzPVD | ZzKTK | ZzPNR | ZzKLZ | ZzAPR | ZzKLR |
| --- | --- | --- | --- | --- | --- | --- | --- | --- | --- | --- |
| ZzKNR | 0 |  |  |  |  |  |  |  |  |  |
| ZzCLT | 0 | 0 |  |  |  |  |  |  |  |  |
| ZzPZR | 0.0372 | 0.036 | 0 |  |  |  |  |  |  |  |
| ZzKLD | 0.99896 | 0.99896 | 0.99946 | 0 |  |  |  |  |  |  |
| ZzPVD | 0.99517 | 0.99517 | 0.99556 | 0.075 | 0 |  |  |  |  |  |
| ZzKTK | 0.4107 | 0.41133 | 0.45442 | 0.70288 | 0.70075 | 0 |  |  |  |  |
| ZzPNR | 0.61873 | 0.61841 | 0.61634 | 0.58341 | 0.57425 | 0.24174 | 0 |  |  |  |
| ZzKLZ | 0.45762 | 0.45757 | 0.46662 | 0.5267 | 0.52575 | 0.17366 | 0.14513 | 0 |  |  |
| ZzAPR | 0.44582 | 0.44658 | 0.45445 | 0.56075 | 0.55699 | 0.20674 | 0.18749 | 0.12196 | 0 |  |
| ZzKLR | 0.5358 | 0.53611 | 0.56058 | 0.63011 | 0.62571 | 0.29305 | 0.27669 | 0.20127 | 0.20207 | 0 |

Mean *F*st = 0.50954 (significant *p <* 0.05)
