# Supplementary material for: Analyses between Reproductive Behavior, Genetic Diversity and Pythium Responsiveness in Zingiber spp. Reveal an Adaptive Significance for Hemiclonality
Source: Front Plant Sci. 2016 Dec 20;7:1913. doi: 10.3389/fpls.2016.01913 (PMC5167741; doi:10.3389/fpls.2016.01913)
Supplement: Supplementary file 2 [file Data_Sheet_1.DOCX]

**Supplementary Figures**

**Figure S1(a)** AFLP fingerprint of 13ginger cultivars revealed by the primer combination E-ACT x M-CTA. Three individuals were analyzed from each cultivar. Name given at the top of the lanes corresponds to Code No. in Table S1.M – 30-330 bp AFLP ladder (Life Technologies)

**
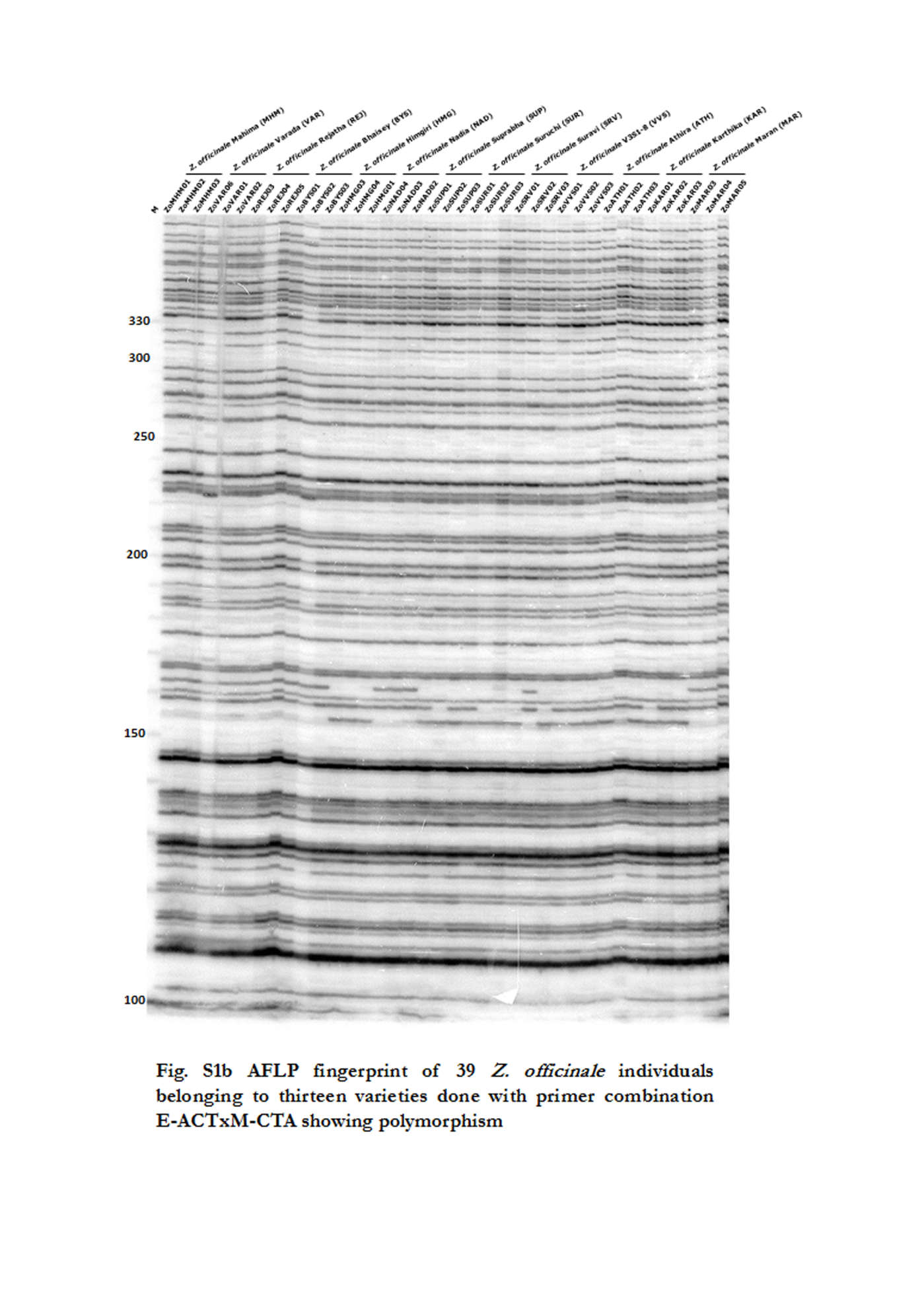
**

**Figure S1(b)** AFLP fingerprint of 13 ginger cultivars revealed by the primer combination E-ACT x M-CTT. Three individuals were analyzed from each cultivar. Name given at the top of the lanes corresponds to Code No. in Table S1.M – 30-330 bp AFLP ladder (Life Technologies).

**
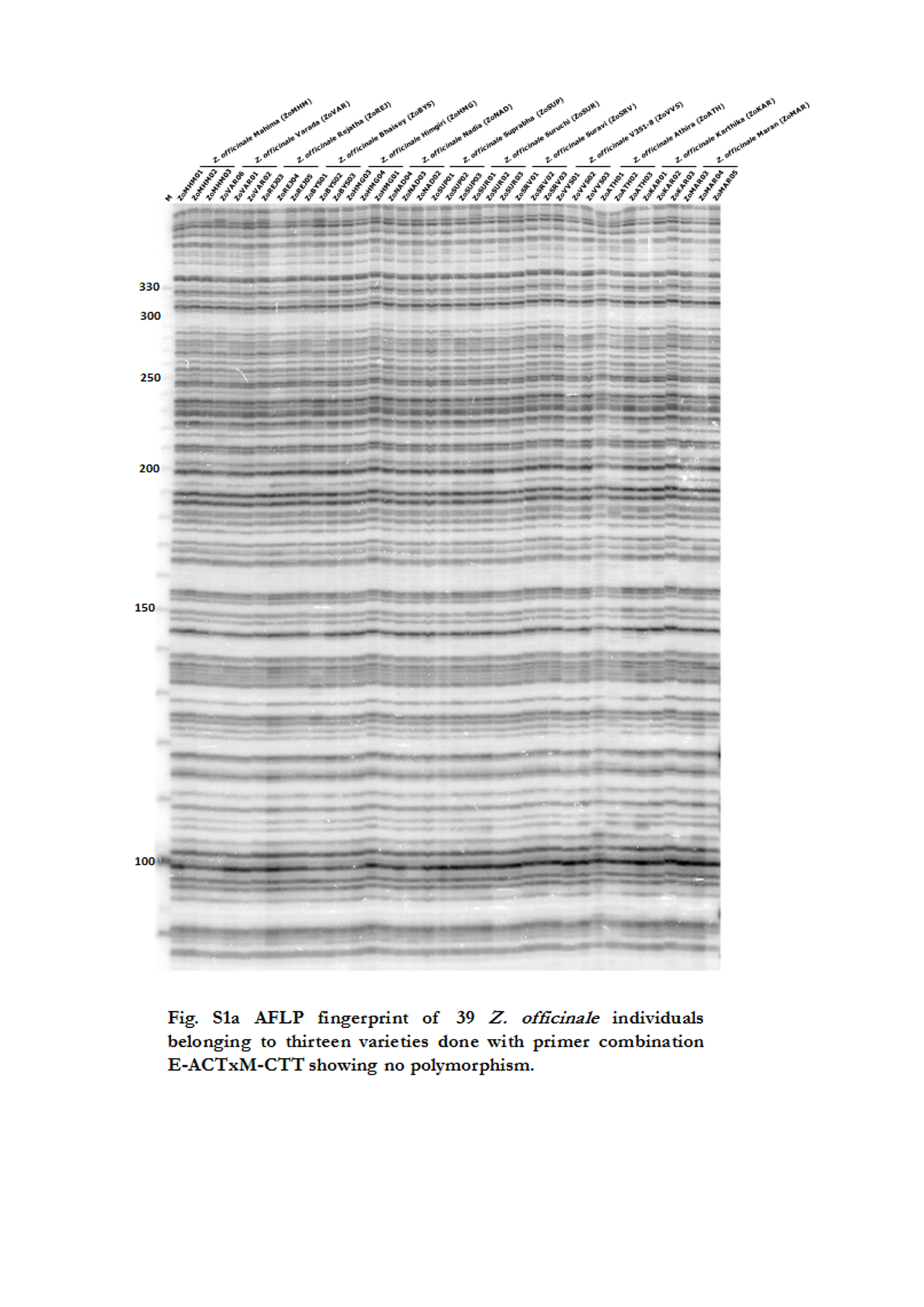
**

**Figure S2** AFLP fingerprint of 30 *Z. neesanum* individuals belonging to three populations revealed by the primer combination E-ACA x M-CTT. Name given at the top of the lanes corresponds to Code No .in Table S2a.M – 30-330 bp AFLP ladder (Life Technologies).


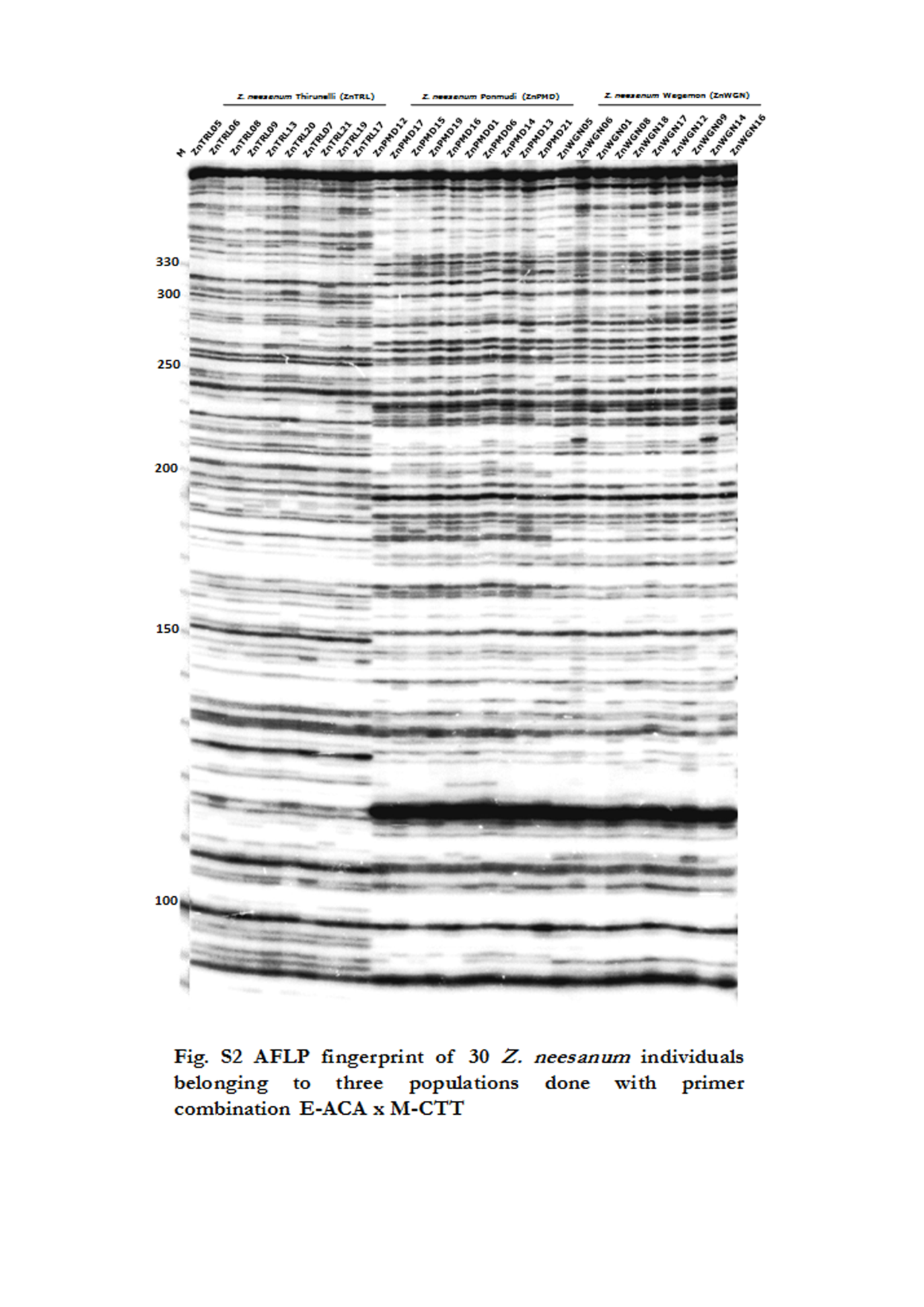


**
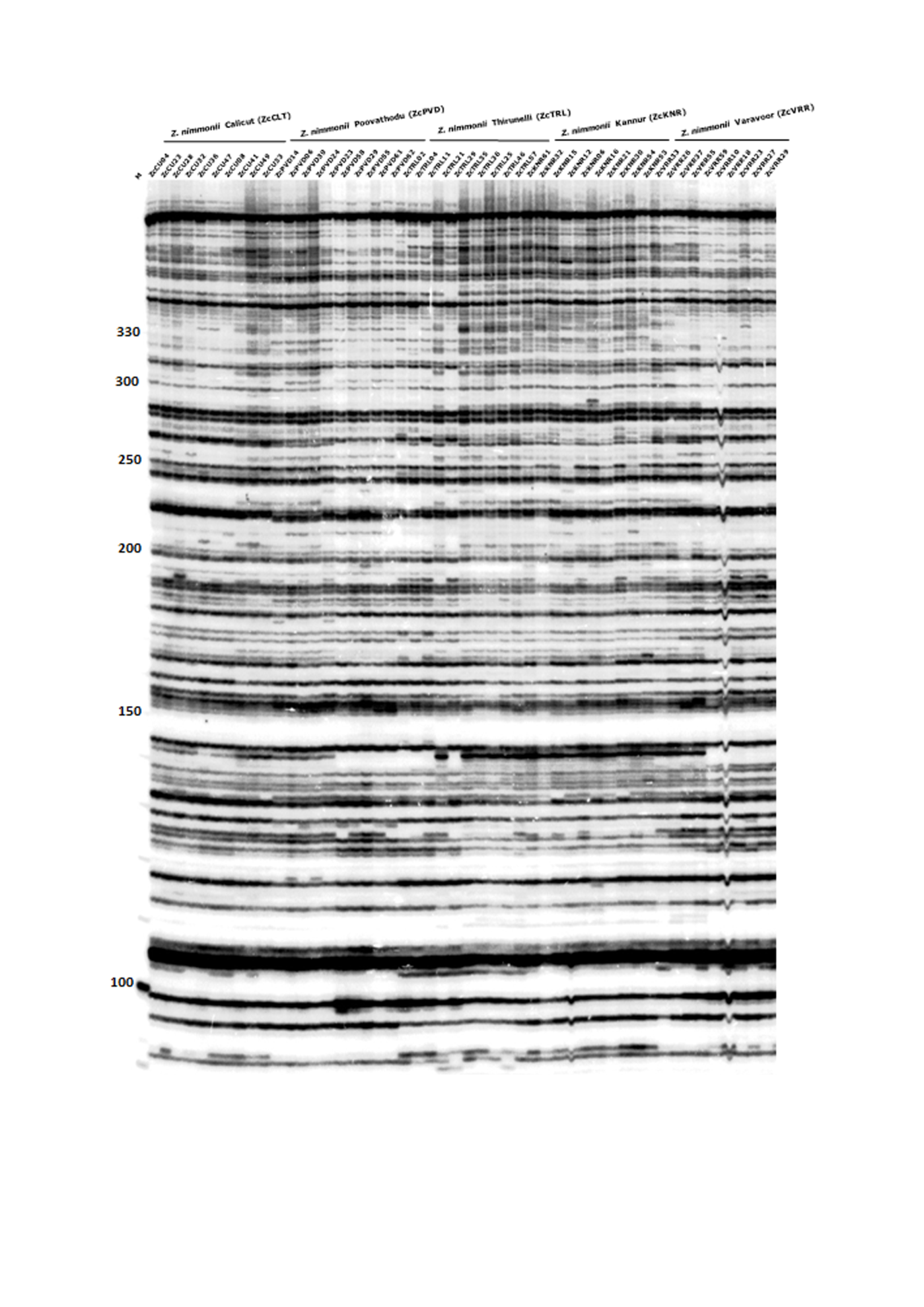
Figure S3** AFLP fingerprint of 50 individuals belonging to five populations of *Z. nimmonii* revealed by the primer combination E-ACA x M-CTT. Name given at the top of the lanes corresponds to Code No.in Table S2b.M – 30-330 bp AFLP ladder (Life Technologies).

**Figure S4(a)** AFLP fingerprint of 70 *Z. zerumbet* individuals belonging to seven populations sampled from revenue land revealed by the E-ACT x M-CTA. Name given at the top of the lanes corresponds to Code No. in Table S2c.M – 30-330 bp AFLP ladder (Life Technologies).

**
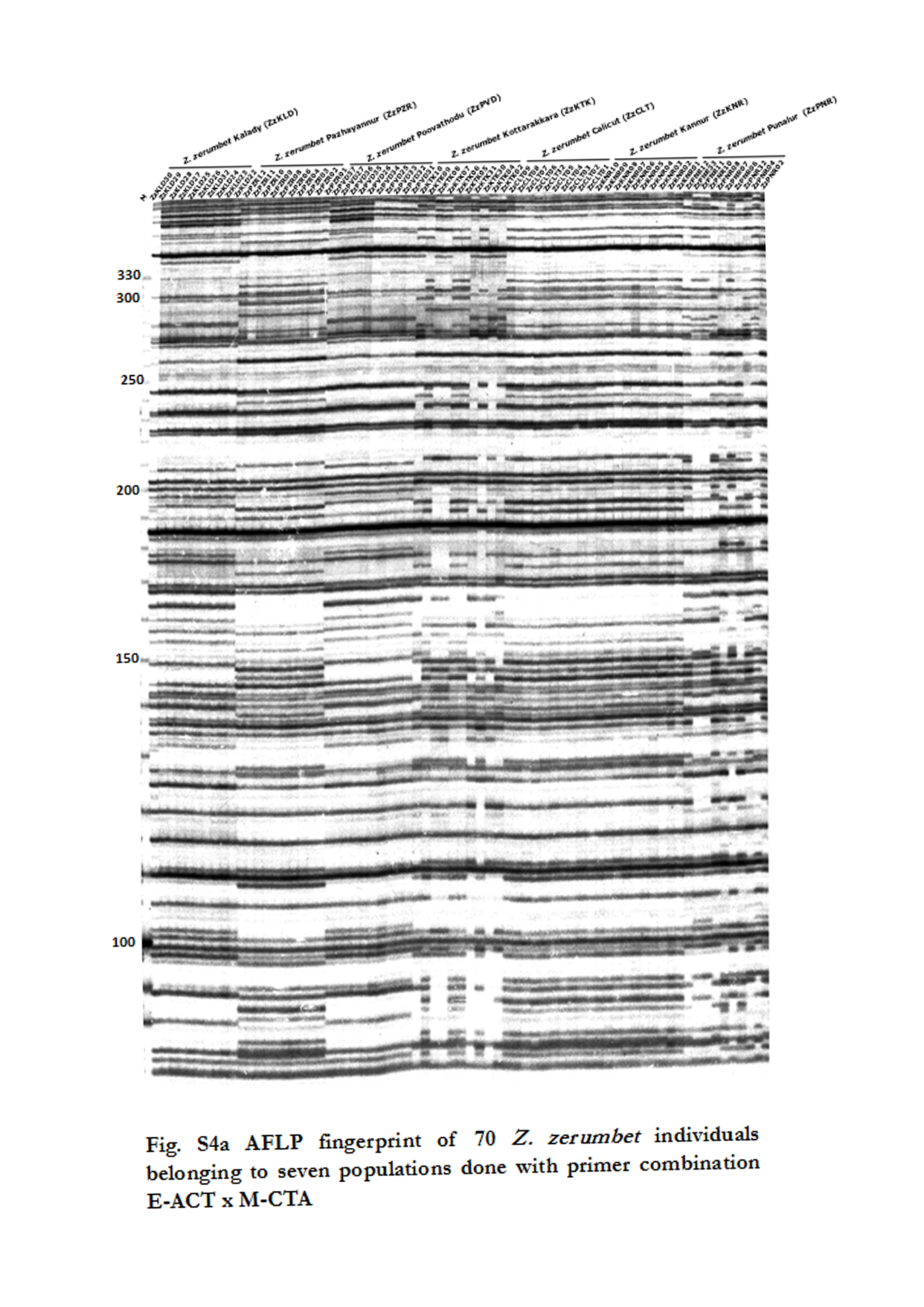
**

**
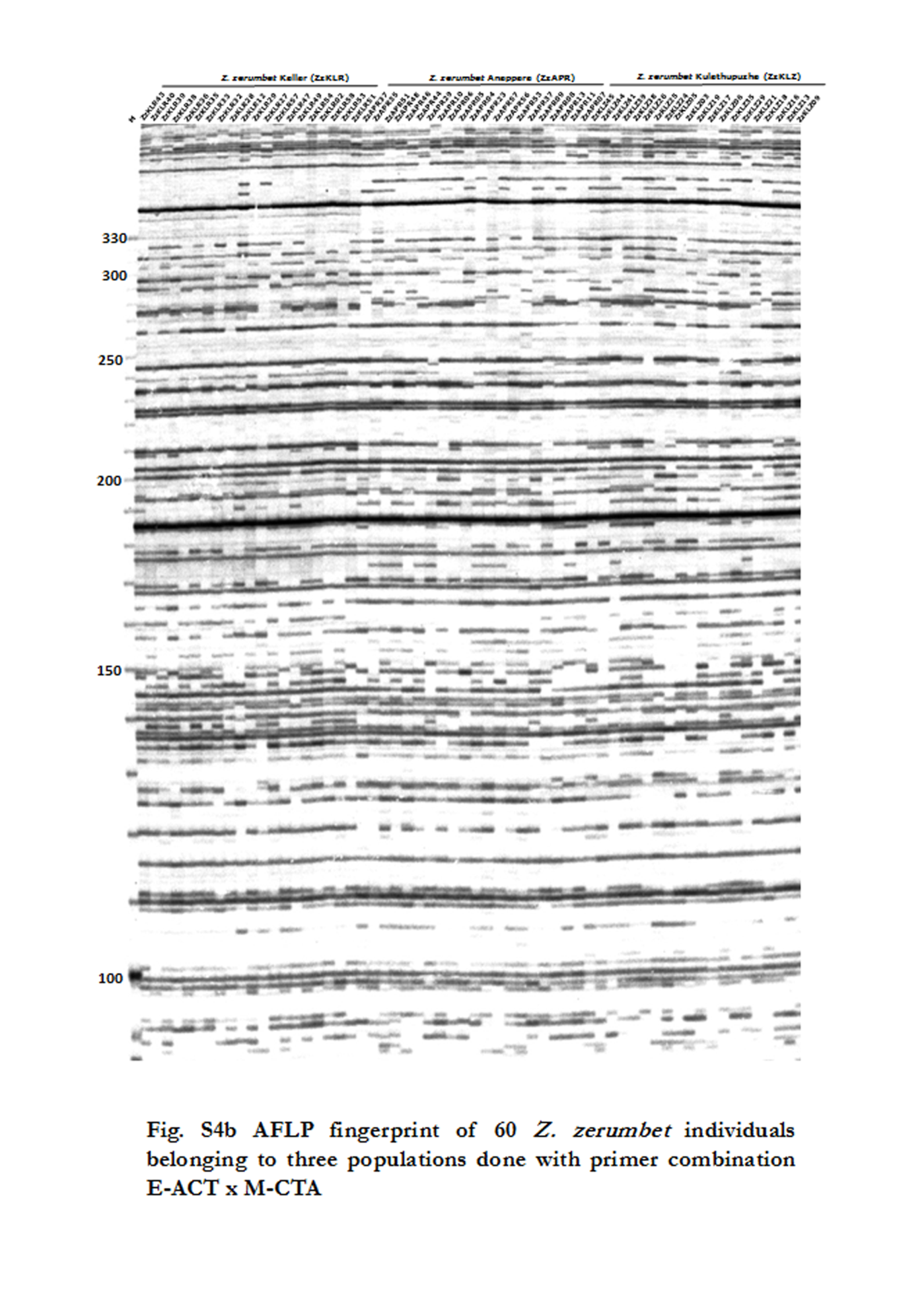
Figure S4 (b)** AFLP fingerprint of 60 *Z. zerumbet* individuals belonging to three populations sampled from forest land revealed by the primer combination E-ACT x M-CTA. Name given at the top of the lanes corresponds to Code No. in Table S2c.M – 30-330 bp AFLP ladder (Life Technologies).

**Figure S5** Population genetic structure of 30 individuals of *Z. neesanum*, 50 individuals of *Z. nimmonii* and 130 individuals of *Z. zerumbet* yielded by STRUCTURE simulations based on AFLP data. **(a)**, **(c)** and **(e)**: Estimating the optimum number of population genetic clusters using ∆*K* values (Y-axis) for *K* ranging from 1-10 (X-axis)in *Z. neesanum*, *Z. nimmonii* and *Z. zerumbet*, respectively using the method proposed by Evanno *et al* (2005).**(b)**, **(d)** and **(f)**: Estimated population structure in *Z. neesanum*(*K* = 3), *Z. nimmonii* (*K* = 4) and *Z. zerumbet* (*K* = 4),respectively. The population genetic clusters identified by the Bayesian algorithm are colour coded and the populations represented in each cluster is indicated at the bottom of the plot using the population name given in Fig. 1. Each individual is represented by a single vertical bar in the cluster and the patches of a different colour in a cluster indicate genetic admixture with the group representing the respective colour code

**
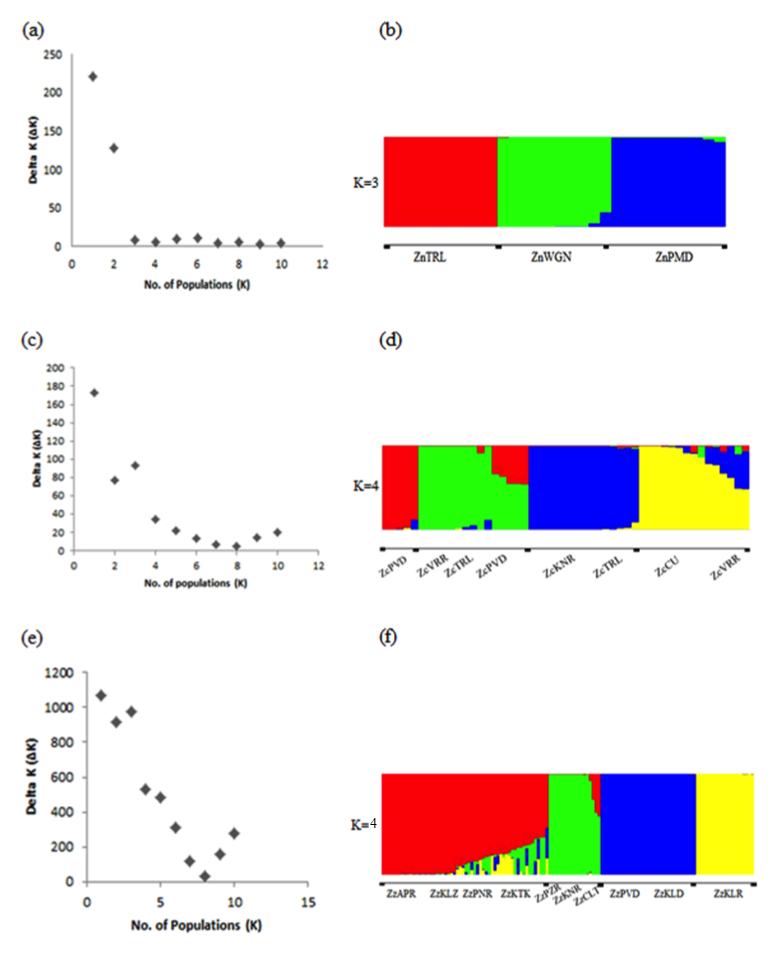
**

**Figure S6** UPGMA dendrogram of 130 individuals belonging to ten populations of *Z. zerumbet* generated by SAHN module of NTSYSpc based on DICE genetic distance computed from AFLP data. Cophenetic correlation *r* = 0.96044 and was determined using the COPH and MXCOMP procedure of NTSYSpc. The name given at the end of the nodes corresponds to code no. in Table S2c

**
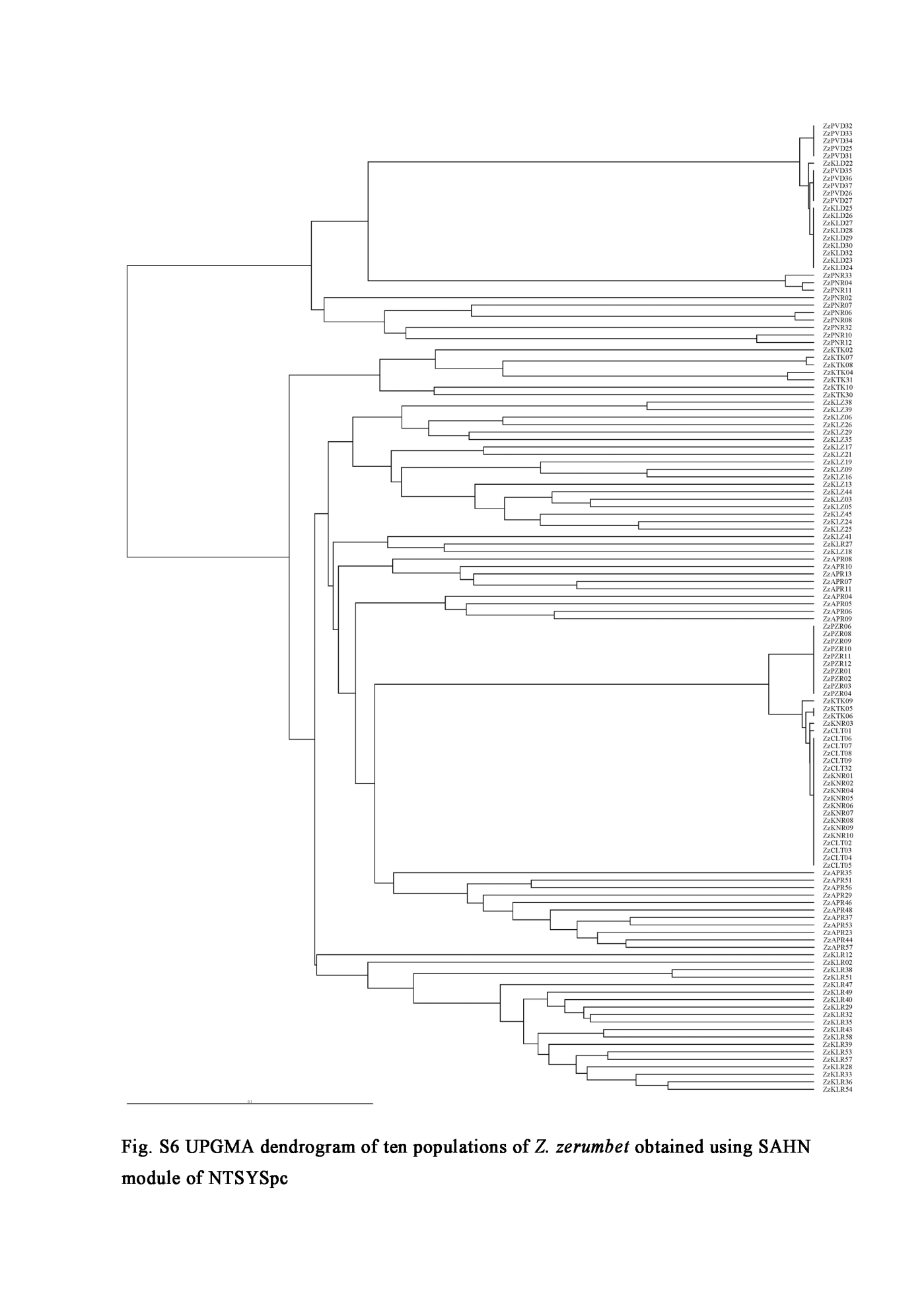
**
